# Development of an advanced flow cytometry based high-resolution immunophenotyping method to benchmark early immune response in dairy cows

**DOI:** 10.1038/s41598-021-02405-w

**Published:** 2021-11-24

**Authors:** Sabine Farschtschi, Martin Mattes, Alex Hildebrandt, Dapi Chiang, Benedikt Kirchner, Heike Kliem, Michael W. Pfaffl

**Affiliations:** 1 Division of Animal Physiology and Immunology, TUM School of Life Sciences, Technical University of Munich, Freising, Germany; 2 TUM School of Life Sciences, Technical University of Munich, Freising, Germany

**Keywords:** Immunology, Physiology

## Abstract

The determination of the somatic cell count of a milk sample is one of the most common methods to monitor udder health of a dairy cow. However, this procedure does not take into account the fact that cells in milk present a great variety of different cell types. The objective of our study was to establish a high-resolution differential cell count (HRDCC) by means of flow cytometry in blood and milk. We were able to detect ten subpopulations among the three main populations of immune cells and to determine their viability. Additionally, blood samples were analyzed for common laboratory biomarkers, i.e. differential blood counts, haptoglobin levels and several metabolic parameters. In this first feasibility study, we used three different vaccines to stimulate the immune system of five healthy cows each. Samples were collected shortly before, in between and after the vaccinations. Using multivariate statistical methods we saw a diagnostic benefit when HRDCCs were included compared to only the standard laboratory parameters. The impacts of all three vaccinations on the immune system were visible in blood HRDCCs as well as in milk HRDCCs. Cluster of Differentiation 8^+^ (CD8^+^) T cells, B cells and monocyte/macrophage subpopulations were among the most important and statistically relevant parameters for all treatments in both biofluids. Moreover, in one of the treatment groups intermediate monocytes showed a significant increase after both vaccinations. Although the use of HRDCC in blood or milk was shown to be highly relevant for early systemic diagnostic, to confirm these subpopulations further investigations in cows of different breed, lactation stage or health status are required.

## Introduction

The somatic cells that are present in bovine milk consist mainly of leukocytes that have crossed the blood-milk barrier as part of the immune defense. In a much lower amount, mammary gland epithelial cells (MEC) are exfoliated from the udder epithelial tissue and shed into the milk during secretion. The determination of the total somatic cell count (SCC) of a raw milk sample, e.g. within the monthly Dairy Herd Improvement (DHI) testing, is a widely used method to control the udder health of an individual cow or at herd level^1–3^. The leukocytes found in milk include different (sub-)populations of immune cells, each of which performs its own specific function during an immune response, as extensively reviewed by Sordillo^4^. Consequently, it makes sense to take the diverse somatic cell types into account by determining a differential cell count (DCC), e.g. using light microscopy^5^ or flow cytometry^6,7^. The latter method is preferable for immunophenotyping due to higher specificity, efficiency and accuracy. Moreover, the use of fluorochrome-labeled antibodies enables a more detailed and accurate analysis of cell subpopulations^8^. Over the years, various milk DCC-based biomarker were presented that could allow a more precise and early diagnosis of mastitis. Pilla et al.^9^ for example found that a logarithmic neutrophil granulocyte:lymphocyte ratio can be used to diagnose an inflamed mammary gland when it is combined with SCC. Schwarz et al.^10^ assessed the health status of a mammary gland by means of a CD2/CD21 index. Other biomarkers aim at the evaluation of the infection phase^6^ or the distinction between ‘acute mastitis’ and ‘chronic mastitis’^11^. Besides scientific approaches, automated cell counters were invented for the determination of the DCC of raw milk samples. The on-farm analyzer QScout Farm Lab (Advanced Animal Diagnostics Inc., USA) uses image cytometry to record a three-part DCC including the proportions of neutrophils, lymphocytes and macrophages. A far bigger analysis capacity of up to 600 samples per hour can be measured by Fossomatic DC (FOSS Analytical A/S, Denmark) by means of flow cytometry^12^. This machine quantifies the macrophages and the combined proportion of PMNs and lymphocytes in bovine raw milk^13^.

In contrast to the many biomarkers that can be used for mastitis diagnostics, very little is known about milk DCC patterns derived from other systemic cattle diseases that are not directly affecting the mammary gland. The aim of our study was to develop a high-resolution immunophenotyping method in bovine blood and milk, based on advanced flow cytometry, to quantify several immune relevant subpopulations and to assess the systemic immune status in dairy cows, preferable directly from milk, induced by diverse vaccination types.

## Material and methods

### Animal study

The study was performed at the research station Veitshof, School of Life Sciences, Technical University of Munich in Freising, Germany. 15 clinically healthy pregnant Brown Swiss cows between the first and the fifth lactation were implemented in the study. They were on average 246 days in milk (n = 188, CV = 55%) with an average milk yield in the morning of 15.35 L (n = 188, CV = 19%). The cows were housed in a freestall barn with rubber-coated slatted floors and cubicles cushioned with wood shavings and they were milked twice daily in a 2 × 2 tandem milking parlor (GEA WestfaliaSurge GmbH). Water was available ad libitum and all animals were fed in average a daily basic ration of 18 kg corn silage, 14 kg grass silage and 1.5 kg hay, supplemented with 1.5 kg high-protein rape seed and soy bean extraction meal (deuka Kompopur 404, Deutsche Tiernahrung Cremer) to achieve the necessary ruminal nitrogen balance^14,15^. Additionally, 190 g mineral mix (Complett Keragen Longlife, Josera) were added to the diet for an adequate mineral level. Apart from that, cows were fed 0.5 kg concentrated feed (deuka MK 194-UDP, Deutsche Tiernahrung Cremer) per liter of delivered milk to ensure the energy supply required for the respective performance. All animals were kept under optimal conditions in accordance with good agricultural practice. A general veterinary examination was carried out at least on every sampling day and the health status of every cow was documented in detail. This animal study was performed in compliance with the German Animal Welfare Act (TierSchG) and the German regulations on the welfare of animals used for experiments or for other scientific purposes (Tierschutz-Versuchstierverordnung, TierSchVersV). The animal study, its sampling regimen and humane endpoints were approved by the government of Upper Bavaria in Munich, Germany (permit number: ROB-55.2-2532.Vet_03-17-70).

### Immune stimulation by vaccinations

**Figure 1:**
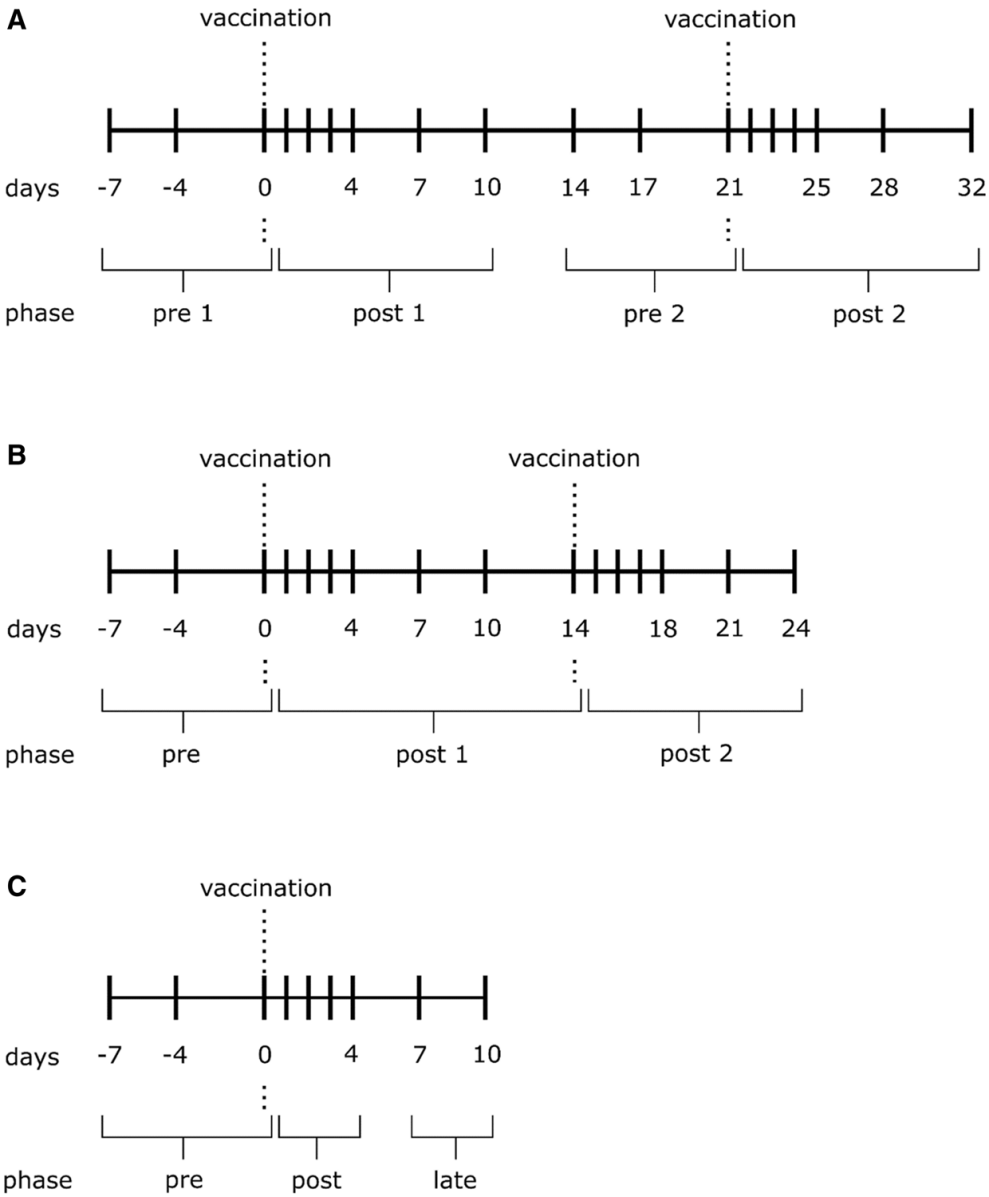
Sampling schemes in three different treatment groups: (**A**) (Bovalto Respi 3), (**B**) (Insol Trichophyton) and (**C**) (Bovela); n = 5 each.

To challenge the immune system and to prove that resulting immune stimulatory effects are visible and measurable by means of our high-resolution immunophenotyping method, three groups of five randomly chosen cows each were vaccinated with approved bovine drugs. The vaccines have never been used on these cows before. Bovalto Respi 3 (Boehringer Ingelheim Vetmedica GmbH) was administered s.c. twice to group A, group B was treated i.m. twice with Insol Trichophyton (Boehringer Ingelheim Vetmedica GmbH) and group C was vaccinated i.m. once with Bovela (Boehringer Ingelheim Vetmedica GmbH). All vaccines were applied by a veterinarian and used as recommended and approved by the manufacturer. Blood and milk samples were taken before and after the immunizations, as shown in Fig. 1.

Group A was vaccinated against the bovine respiratory disease with Bovalto Respi 3. As this disease is multifactorial, the vaccine includes three inactivated strains of the major pathogens, both viral and bacterial, i.e. Parainfluenza 3, Bovine Respiratory Syncytial Virus, and Mannheimia haemolytica. It also contains the adjuvants aluminum hydroxide and Quillaja saponin (Quil A). This immunization leads to production of neutralizing antibodies, stimulation of T helper type 1 cells^16^ and to various other antibody-mediated mechanisms^17^.

Insol Trichophyton, used in group B, contains a combination of microconidia of the fungal strains T. verrucosum, T. mentagrophytes and T. sarkisovii that were inactivated in an early germination phase. This vaccination results primarily in an activation of T helper type 1 cells^18,19^.

Group C was immunized with Bovela, which consists of two genetically modified strains of live bovine viral diarrhea virus, BVDV type 1 and 2, with deletions of the E^rns^ and N^pro^ genes^20^. This vaccine does not induce lymphopenia and is able to stimulate a response of memory CD4, CD8 and gamma delta T cells, as shown by Platt et al.^21^ using CD25 as an activation marker.

### Milk sampling and cell isolation

Cow composite milk samples of all four quarters were collected in the milking machine evenly throughout the whole milking process of each cow in the morning. Since the samples were directly brought to the laboratory for subsequent processing, no preservative was added. Isolation of milk cells was conducted according to the modified protocol described by Koess and Hamann^22^. Milk samples were thoroughly mixed by inverting five times and then subdivided into four aliquots of 25 ml each. Subsequently, aliquots were diluted 1:2 with cold DPBS (Dulbecco’s Phosphate Buffered Saline, Sigma Aldrich, Co.). After the first centrifugation (15 min, 1000×*g*, 4 °C) the fat layer was removed and the supernatant was poured off in each aliquot. The remaining cell pellets were resuspended with 3.5 ml DPBS each and all four suspensions were combined into a single tube prior to subsequent centrifugation (10 min, 400×*g*, 4 °C). Afterwards, the supernatant was discarded and the cell pellet was washed with 8 ml DPBS and centrifuged again (10 min, 400×*g*, 4 °C). The supernatant was discarded once again before the final cell pellet was resuspended in 1 ml DPBS. The samples were kept on ice until further processing.

### Blood sampling and cell isolation

Blood samples were taken by a veterinarian after morning milking. Peripheral blood was obtained from the jugular vein into a 9 ml EDTA pre-coated Vacuette tube and a 4 ml Serum Vacuette tube with a comprised clot activator (Greiner Bio-One GmbH). Samples were directly brought to the laboratory for immediate processing. 3 ml EDTA blood were diluted with 8.5 ml cold ACK (Ammonium-Chloride-Potassium) lysis buffer (0.15 M NH_4_Cl, 13 mM KCl, 0.1 mM Na_2_EDTA, pH 7.4, sterile filtered) and incubated on ice for 10 min. The cell pellet was obtained by centrifugation (10 min, 400×*g*, 4 °C) and washed two more times with 4 ml cold ACK buffer. After the last centrifugation step the supernatant was poured off and the cell pellet was resuspended in 1 ml cold DPBS.

### Cell staining

Cell numbers of milk and blood cell suspensions were determined with a TC10 Automated Cell Counter (BioRad Laboratories Inc.), and further recounted randomized using a Neubauer Chamber (Hecht Assistent). Subsequently, 10^6^ milk or blood cells per sample were transferred into separate tubes and 1 ml cold FACS buffer (DPBS with 2% fetal bovine serum (Sigma Aldrich, Co.) and 0.01% NaN_3_) was added. Then cell samples were centrifuged (5 min, 500×*g*, 4 °C) and supernatants were poured off. Meanwhile mastermix 1 (Supplementary Table S1) containing DPBS and the cell viability dye was prepared. 100 µl of cold mastermix 1 were pipetted onto every cell pellet and the pellets were resuspended. Suspensions were incubated in the dark on ice for 30 min. Afterwards, 1 ml cold FACS buffer was added to every cell suspension and the samples were centrifuged (5 min, 500×*g*, 4 °C). Supernatants were then poured off and cold mastermixes 2, 3 and 4 (Supplementary Table S1) were pipetted separately onto different cell pellets and resuspended. Those three mastermixes contained FACS buffer and primary antibodies for the three different color panels to quantify cell sub-populations. In addition, 6 mastermixes with isotype control antibodies (Supplementary Table S2) were used as FMO (fluorescence minus one) controls when applicable. The cell suspensions were incubated on ice and protected from light for 30 min. Thereafter, 1 ml cold FACS buffer was added to each sample, samples were centrifuged (5 min, 500×*g*, 4 °C) and supernatants were discarded. Then cell pellets were resuspended in 1 ml cold FACS buffer, samples were centrifuged again (5 min, 500×*g*, 4 °C) and supernatants subsequently discarded. In the last staining step cell pellets were resuspended in the corresponding cold secondary antibody mastermixes, mastermixes 5 or 6 (Supplementary Table S1) respectively, that had been prepared before. In addition, unspecific binding of secondary antibodies was ruled out by using negative controls without primary antibodies. The following incubation on ice lasted 30 min. After that, 1 ml cold FACS buffer was pipetted on every cell suspension prior to centrifugation (5 min, 500×*g*, 4 °C). Supernatants were discarded, cell pellets resuspended in 1 ml cold FACS buffer and centrifuged again (5 min, 500×*g*, 4 °C). After the supernatants were poured off, cell pellets were ready for the ensuing sample fixation.

### Sample fixation

Stained cell pellets were resuspended in 200 µl cold fixation buffer (DPBS with 0.03% NaN_3_ and 1% CH_2_O) and incubated on ice, protected from light for 15 min. Subsequently, 1 ml cold FACS buffer was added to each of the cell suspensions, samples were centrifuged (10 min, 1000×*g*, 4 °C) and supernatants then discarded. Lastly, the cell pellets were resuspended in 120 µl cold FACS buffer and stored in the dark at 4 °C for a maximum period of 24 h.

### Flow cytometric analysis

Prior to the flow cytometric analysis, all samples were filtered through a 100 µm mesh to remove clumps and prevent the flow cytometer from clogging. The analysis was performed with a BD LSRFortessa flow cytometer (Becton, Dickinson and Company) equipped with four lasers and the appendant BD FACSDiva software. To obtain reliable and stable results, the recommended BD CS&T Research Beads (Becton, Dickinson and Company) were used before every run. Additionally, a thorough compensation was performed for all antibody panels before every experiment using the AbC Total Antibody Compensation Bead Kit (Thermo Fisher Scientific Inc.) to correct the emission spectra overlap of the fluorochromes. For milk cells, an average of 468,843 events (n = 430, CV = 38%) was recorded and, for blood cells, 286,369 events (n = 430, CV = 21%) were acquired on average.

### Gating strategy

**Figure 2:**
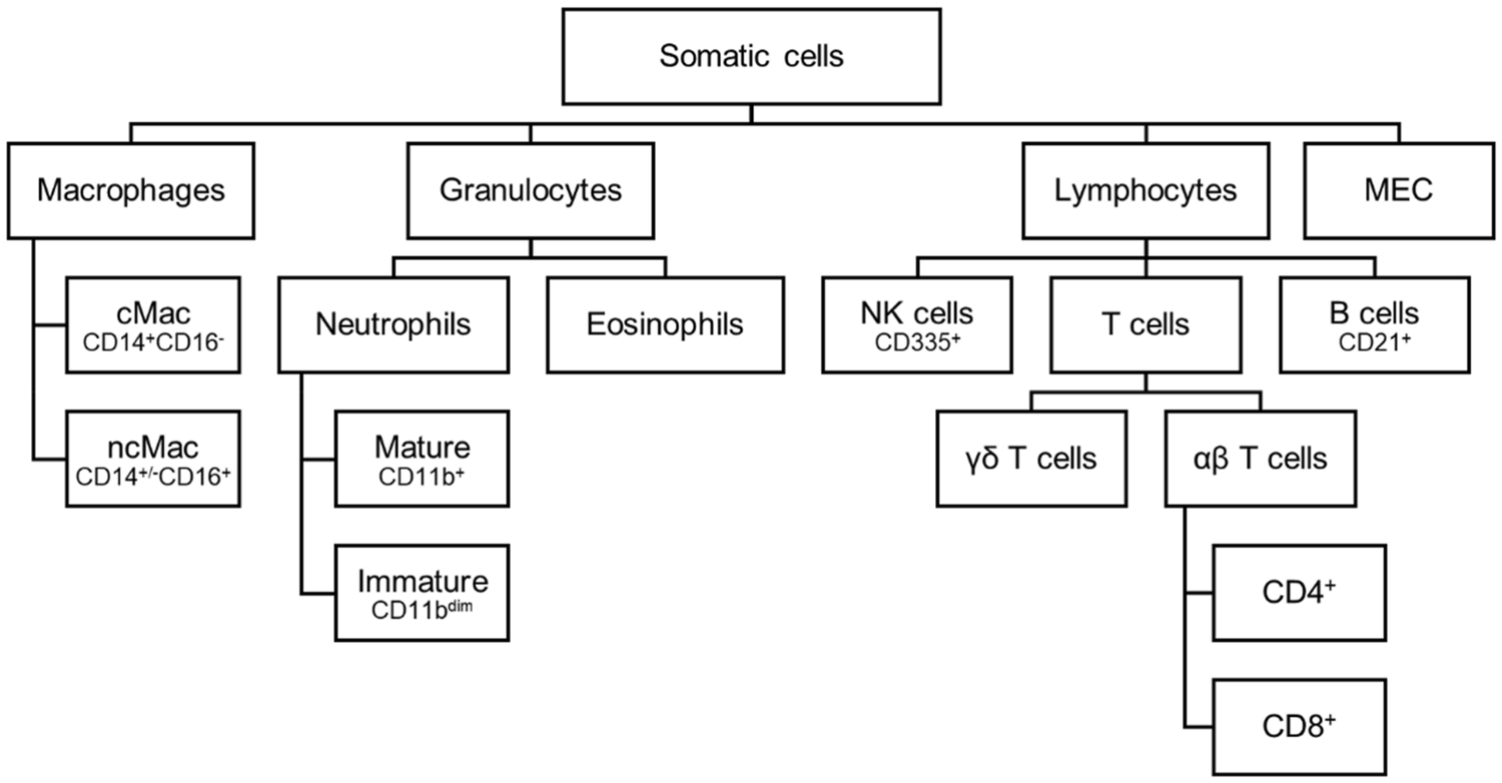
Overview of the detected cell populations in milk.

**Figure 3:**
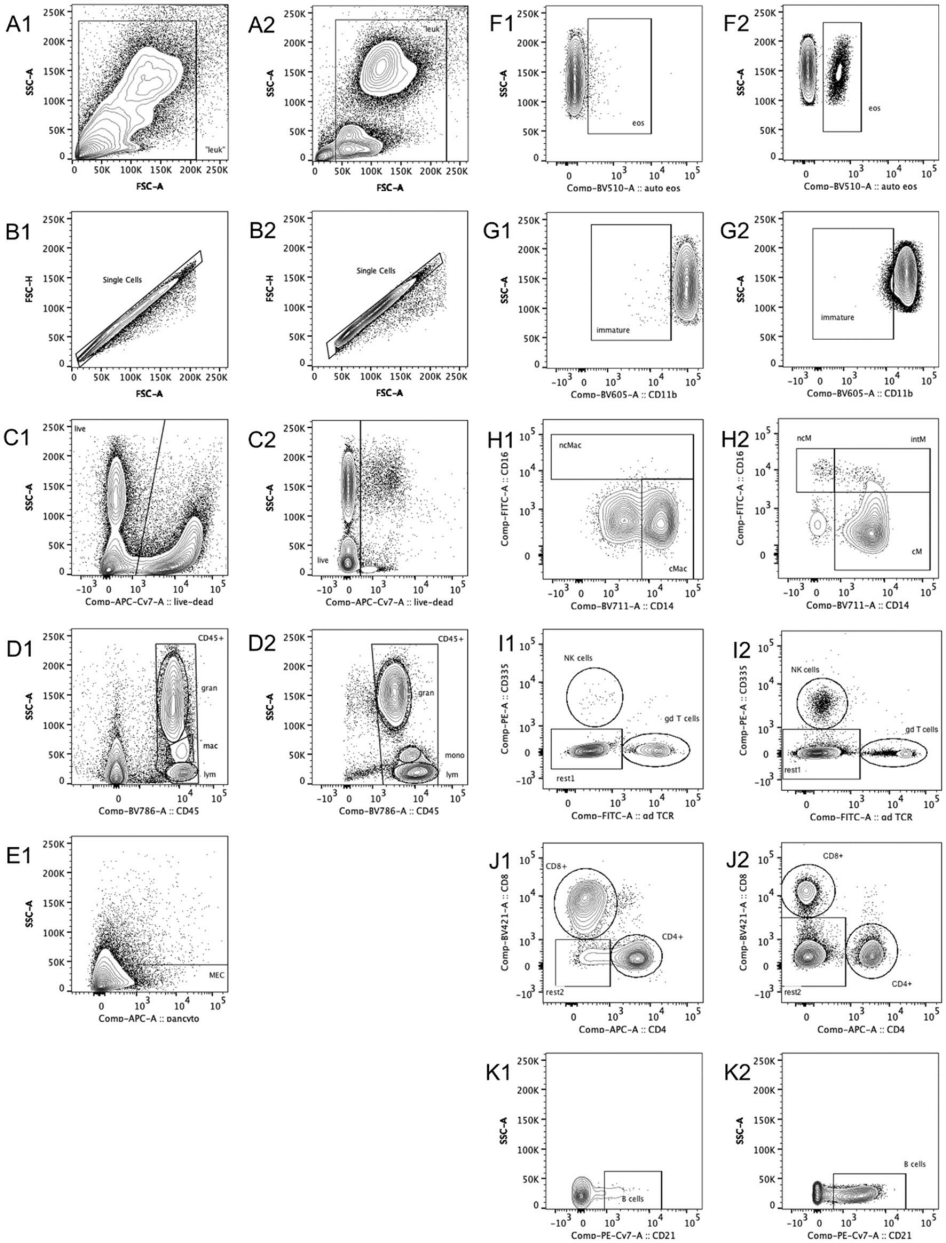
Gating strategy. (**A**) FSC-A vs. SSC-A, milk cells (A1), blood cells (A2); (**B**) Doublet discrimination, milk cells (B1) and blood cells (B2); (**C**) Determination of viability, single cells, milk (C1) and blood (C2); (**D**) Pan leukocyte marker CD45 vs. SSC-A, live cells, milk (D1) and blood (D2); (**E1**) Pan cytokeratin marker vs. SSC-A, CD45^-^ cells, milk; (**F**) Autofluorescence (filter 525/50 nm) vs. SSC-A, granulocytes, milk (F1) and blood (F2); (**G**) CD11b vs. SSC-A, granulocytes, milk (G1) and blood (G2); (**H**) CD14 vs. CD16, monocytes/macrophages, milk (H1) and blood (H2); (**I**) gdTCR vs. CD335, lymphocytes, milk (I1) and blood (I2); (**J**) CD4 vs. CD8, rest1, milk (J1) and blood (J2); (**K**) CD21 vs. SSC-A, rest2, milk (K1) and blood (K2).

Raw data was evaluated using FlowJo software 10.7.1 (Becton, Dickinson and Company) to discriminate and quantify leukocyte subpopulations as well as MEC and measure their viability (Fig. 2). For all antibody panels (blood and milk) leukocyte populations were first roughly gated to eliminate most of the debris (Fig. 3A). Then, to exclude doublets, single cells were gated by comparing FSC-Area and FSC-Height to each other (Fig. 3B). Afterwards, we distinguished between live and dead cells (Fig. 3C) by using Zombie NIR Fixable Viability Kit (Biolegend, Inc.). CD45, a well-known pan leukocyte marker^23^, was then plotted against SSC-A. Since the SSC values provide information about the granularity of a cell^8^, this allowed us to differentiate the CD45^+^ cells based on their SSC value in granulocytes, macrophages/monocytes or lymphocytes (Fig. 3D).

In the 6 color run (milk), all CD45^-^ cells were further examined using a pan cytokeratin marker to identify mammary gland epithelial cells (Fig. 3E1).

In the 5 color run (blood) and the 6 color run (milk), we further analyzed the cells of the granulocyte-gate (CD45^+^SSC^high^) using the autofluorescence of unstained eosinophils shown in a vacant channel (filter 525/50 nm) upon excitation with a violet laser (405 nm) (Fig. 3F). Thereby, we took advantage of the fact that bovine eosinophils exhibit a bright autofluorescence, which was also shown for human eosinophils^24^, due to granule-associated flavin adenine dinucleotide molecules within the cells^25^.

We furthermore examined the expression of CD11b on granulocytes (Fig. 3G). This membrane adhesion molecule is described to be expressed on granulocytes of all maturation stages, but its frequency of occurrence increases during granulopoiesis^26^. Thus, the fluorescence intensity of CD11b^+^ granulocytes provides information about their stage of maturation and can thereby help to detect immature granulocytes, which could indicate an early inflammation response.

In the 5 color run (blood), we took a closer look at the cells in the monocyte-gate (CD45^+^SSC^mid^) (Fig. 3H). We therefore examined monocyte subsets for their expression of CD14 and CD16 as recommended by Hussen et al.^27^ to identify their subsets of classical monocytes (cM) CD14^+^CD16^-^, intermediate monocytes (intM) CD14^+^CD16^+^ and nonclassical monocytes (ncM) CD14^-^CD16^+^. Similarly, we tried this gating strategy for the cells in the macrophage-gate (CD45 + SSC^mid^) in the 6 color run (milk) to divide them into classical macrophages (cMac) CD14^+^CD16^-^ and nonclassical macrophages (ncMac) CD14^+/-^CD16^+^.

In both 7 color runs (milk and blood), we thoroughly analyzed the cells in the lymphocyte-gate (CD45^+^SSC^low^). We checked the expression of CD335 (NKp46), a natural cytotoxicity receptor on bovine natural killer (NK) cells^28^, to determine this subpopulation (Fig. 3I). We also investigated the percentage of cells positive for gamma delta T-cell receptor (gdTCR). The remaining lymphocytes (CD335^-^gdTCR^-^) were examined for the expression of CD4 and CD8 (Fig. 3J). Subsequently, the rest of the lymphocytes (CD335^-^gdTCR^-^CD4^-^CD8^-^) was further analyzed for the expression of CD21, which is specific for bovine B cells^29^ (Fig. 3K).

### Comparison of the conducted manual gating with an automated gating technique

To reduce the subjectivity that inevitably accompanies manual gating and enhance reproducibility in this highly complex immunophenotyping study, all samples were also evaluated using the automated gating technique of the R package openCyto^30^. This package is able to automatically create gates based on the scheme of the original manual gates. Subsequently, the results of manual and automated gating were compared using the flowWorkspace package^31^ to determine the level of accuracy of the automatic gating and the accordance between manual and automatic gating strategy.

### Additional analysis of blood samples by an external laboratory

In addition to our analysis EDTA blood and serum samples were sent to an external veterinary laboratory (Laboklin GmbH & Co. KG, Bad Kissingen, Germany) for routine measurement screening. By means of multiple photometric assays using the Cobas c701 system (F. Hoffmann-La Roche Ltd) the levels of the following important immune relevant protein biomarkers were determined. This included the level of haptoglobin, one of the most important acute phase proteins in cattle^32^. Furthermore, levels of calcium and of the metabolic parameters β-hydroxybutyric acid (β-HBA), non-esterified fatty acids (NEFA), bilirubin and glutamate dehydrogenase (GLDH) were analyzed. Additionally, a differential blood count was performed using the ADVIA 2120i (Siemens Healthcare GmbH) to allow comparison with the results obtained by our high-resolution immunophenotyping method for granulocytes, monocytes, lymphocytes and eosinophils. Furthermore, the standard differential blood count includes a total leukocyte and erythrocyte count and the amount of band neutrophils. Hematocrit and hemoglobin levels were not considered in the statistical analysis as they strongly relate to the erythrocyte count.

### Statistical analysis

To analyze and compare the quantified parameters, Prism 9.0.0 (GraphPad Software) was used to run a linear regression analysis and a Bland–Altman concordance analysis to compare manual and automated gating results and to compare our results to those acquired by the external laboratory. A Friedman test was also performed with Prism to examine the shift in the percentages of the intM in Group A. P values < 0.05 were considered significant.

Furthermore, multivariate statistical analysis was applied, using unsupervised and supervised clustering techniques to explore the measured data and to discover implicit links between the quantified subpopulations and the treatment groups or phases. Therefore, sampling dates of each vaccination group were assigned to different treatment phases, as shown in Fig. 1, in order to see if the immune reaction can be detected by our selected parameters and if the treatment phases can be distinguished with our applied methods. For group A the phases “pre1” and “pre2” were combined as “pre”, as well as “post1” and “post2” as “post”, since we did not find any significant differences between the fused groups. An unsupervised hierarchical clustering (HC) and a principal component analysis (PCA) was performed first, using Base R^33^ and the R package Factoextra^34^.

Finally, a supervised sparse partial least squares-discriminant (sPLS-DA) and a partial least squares-discriminant (PLS-DA) analysis, both included in the R package mixOmics^35^, were applied to find the most discriminating parameters (herein milk and blood subpopulations) between treatment phases and test these for their predictive value via a receiver operating characteristic curve analysis (ROC curve). The parameters of the blood and milk cell population resulting from our flow cytometric approach and those acquired by the external laboratory were analyzed separately as well as combined.

## Results

### Animal study

Apart from common side effects of the vaccinations, all cows except one remained healthy for the duration of the experiment. The diseased cow from group B, which developed a mastitis after the second vaccination, was properly treated by the veterinarian and therefore excluded from the clustering analysis. All cows from group A and group C showed a slight, superficial swelling at the injection site, which is a known side effect according to the manufacturer. In group B, three of the remaining cows had an increase in body temperature of 1–1.5 °C the day after the vaccination, but no clinical symptoms. The data of two cows, one from group A and one from group C, was not included in the evaluations as the anti-CD45 antibody used in our panel failed to mark the leukocyte populations of the respective cows.

### Consistency between the manual and the automated gating technique

The comparison of the gates that were manually set with the automatically created gates showed a significant agreement in the resulting data for all analyzed cell types and animals. Nearly 5,000 gating comparisons in all three treatment groups were evaluated with a linear regression analysis (n = 4,888, r = 0.99, P < 0.0001).

### Comparison of our results with the externally analyzed differential blood counts

The results we obtained with our high-resolution immunophenotyping method were not overall consistent compared with the differential blood count performed at the external laboratory. After performing a linear regression comparison analysis, the level of correlation varied for the different cell types (granulocytes: n = 183, r = 0.85, P < 0.0001; monocytes: n = 183, r = 0.28, P < 0.0001; lymphocytes: n = 183, r = 0.82, P < 0.0001; eosinophils: n = 183, r = 0.77, P < 0.0001). The satisfactory agreements between the externally analyzed cell count and our own results for granulocytes and lymphocytes were also visible when evaluating the corresponding Bland–Altman plots (Supplementary Figs. 1 and 2). The respective plots for monocyte and eosinophil counts (Supplementary Figs. 3 and 4) showed a less good concordance.

### Shift in percentages of intermediate monocytes after treatments

**Figure 4:**
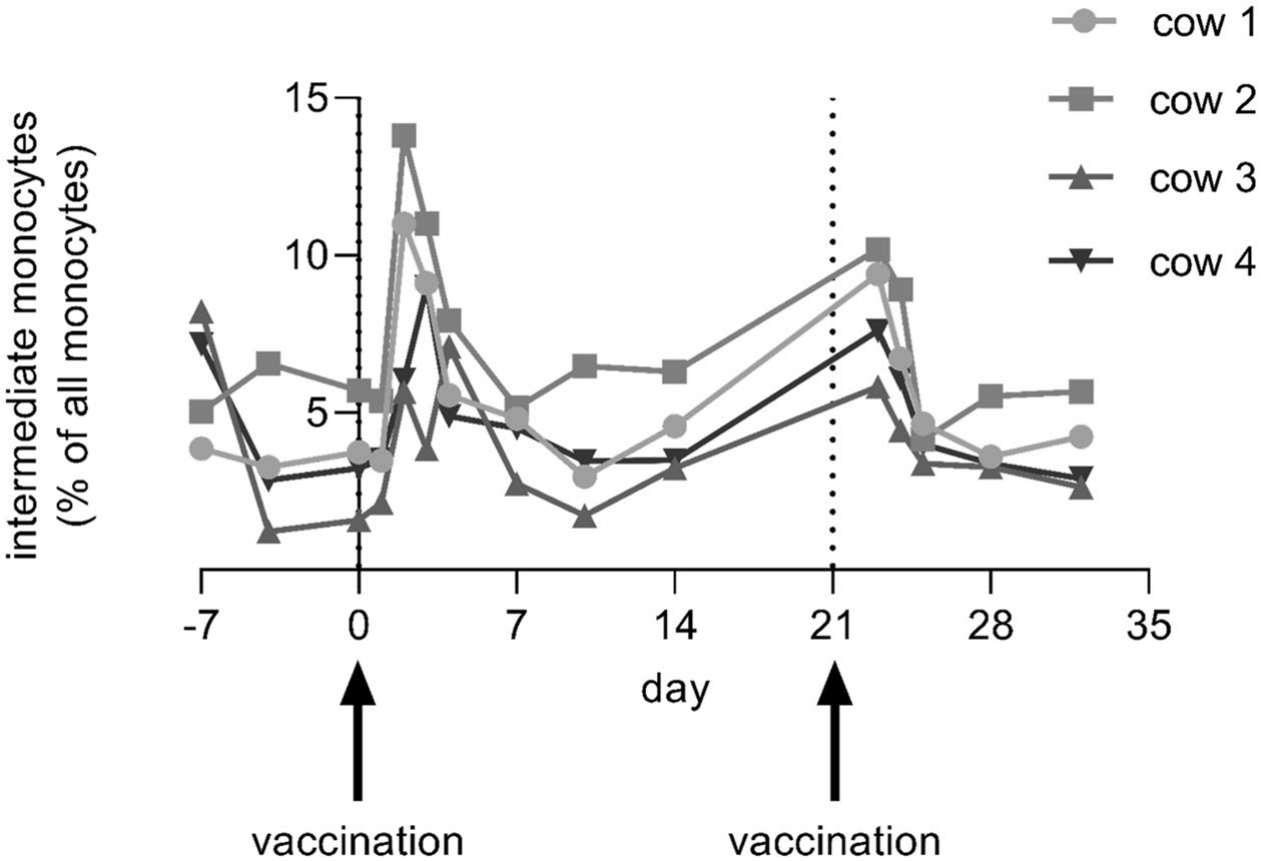
Intermediate monocytes concentration over time in four cows of group A using Bovalto Respi 3 vaccination.

Two to three days after each vaccination using Bovalto Respi 3, the intermediate monocytes of all cows in the group A showed a clear increase in their percentage values (Friedman test, P < 0.001). The shift was more pronounced after the first treatment than after the second, see Fig. 4. Such an effect could not be observed in the other two treatment groups B and C.

### Unsupervised clustering

To reduce complexity and to find the most significant biomarker to predict changes in the immune relevant subpopulations and in the bovine immune status in general, unsupervised and supervised clustering methods were applied. After performing unsupervised PCA and HC, a separation of the treatment phases was noticeable to varying degrees. The cell counts used for clustering are shown in supplement data.

In group A there were only slight differences between the compared phases “pre” and “post” visible when the PCA was performed using only the HRDCC blood parameters. The main influence factors in PC1 were the viability of all leukocytes and the percentage of the total lymphocytes population and in PC2 the percentage of gamma delta T cells and CD8^+^ T cells. When all parameters were taken into account, the clusters of the phases overlapped. A similar outcome could be observed when the PCA was based on only the milk parameters or on only the parameters that were acquired by the external laboratory. This was also true for the HC.

The clusters of the phases of group B differed slightly from each other, in the PCA as well as in the HC analysis. For this group the parameters explaining most of the variance on PC1 were the percentage of milk granulocytes and milk lymphocytes and on PC2 the number of erythrocytes.

In group C the phase “late” could be clearly distinguished from the other two, especially when all parameters were used for the PCA as well as for the HC. This separation was mainly explained in PC1 by the percentage of blood intermediate monocytes and the percentage of blood CD8^+^ T cells and in PC2 by the percentage of milk eosinophils. All PCA and HC graphical results are shown in the supplement data.

### Supervised clustering

**Figure 5:**
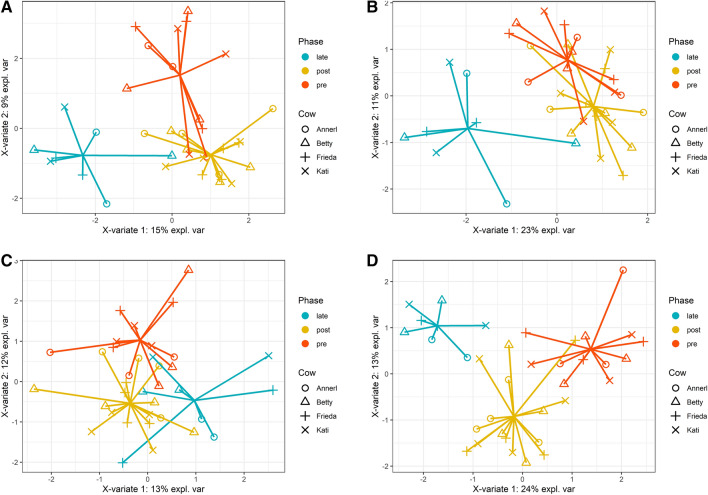
Supervised Clustering of the results of Bovela treatment (group C), three components with a maximum of five features each. (**A**) sPLS-DA using all parameters (AUROC: “pre” vs others: 0.997, “post” vs others: 0.950, “late” vs others: 1.000); (**B**) sPLS-DA using blood parameters (AUROC: “pre” vs others: 0.896, “post” vs others: 0.847, “late” vs others: 0.987); (**C**) sPLS-DA using milk parameters (AUROC: “pre” vs others: 0.931, “post” vs others: 0.906, “late” vs others: 0.973); (**D**) sPLS-DA using the parameters analyzed by external laboratory (AUROC: “pre” vs others 0.979, “post” vs others: 0.959, “late” vs others: 1.000).

The goal of the supervised clustering or classification is to find the most discriminating parameters, herein biomarkers that separate the different treatment phases. Therefore, a sPLS-DA with three components with a maximum of five features each was applied and the different treatment phases were defined as the separating factors. The Bovela treatment (group C) showed the most consistent results. The sPLS-DA was calculated for different parameter sets. For all data sets, a clean separation of all phases (pre, post and late) could be shown. Best AUROC (Area Under the Receiver Operating Characteristics) results were obtained using all parameters available as well as for the biomarker data based on the parameters measured by the external laboratory, followed by “only blood” and “only milk” parameters (Fig. 5).

### Prominent common biomarker subpopulations

**Figure 6:**
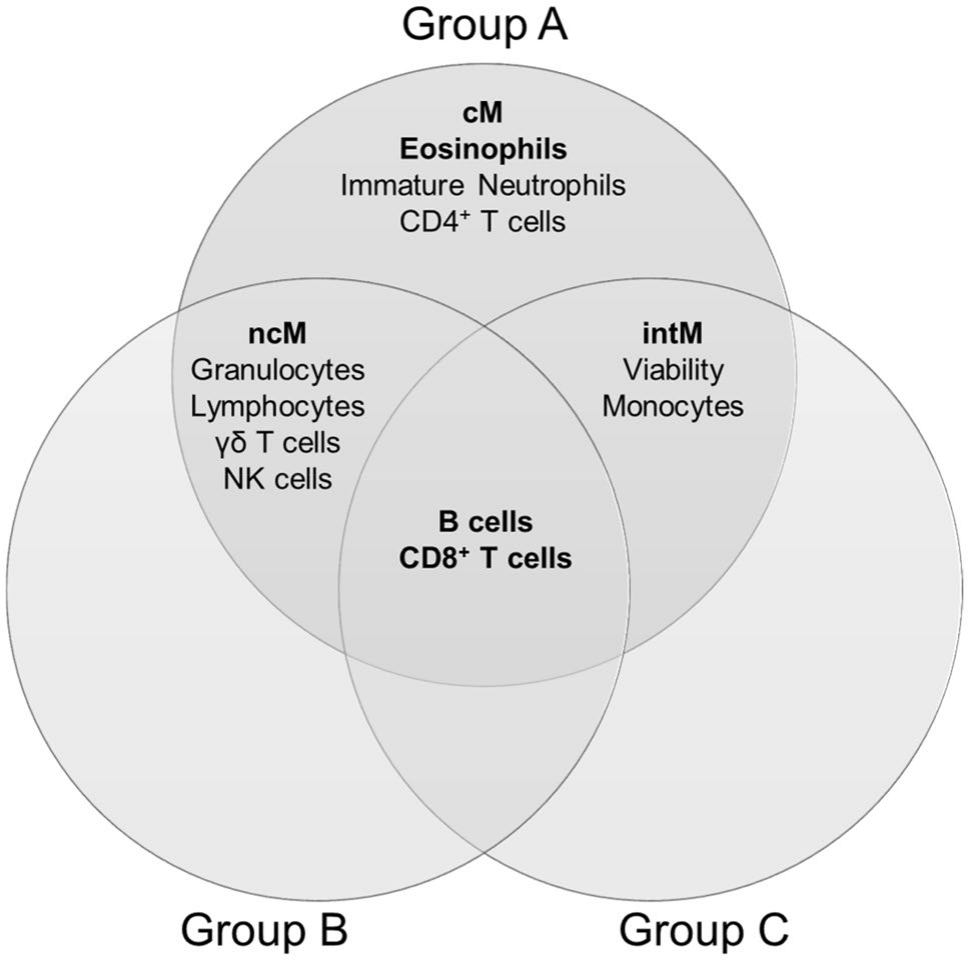
Overview of the most discriminating subpopulations or features of sPLS-DA based on only blood parameters, to separate the treatment phases.

Comparing all three treatments and selecting the most discriminating parameters to separate the treatment phases and hence the immune stimulation, only a few interesting subpopulations appeared for blood and milk. The most important statistically relevant separating subpopulations for all groups were the CD8^+^ T cells and B cells (highlighted in Fig. 6). Besides those, ncM, gamma delta T cells and NK cells as well as the total granulocyte population and the total lymphocyte population influenced the phase differentiation in groups A and B. The intM, the total monocyte population and the overall viability were also responsible for the separation of the treatment phases in groups A and C.

**Figure 7:**
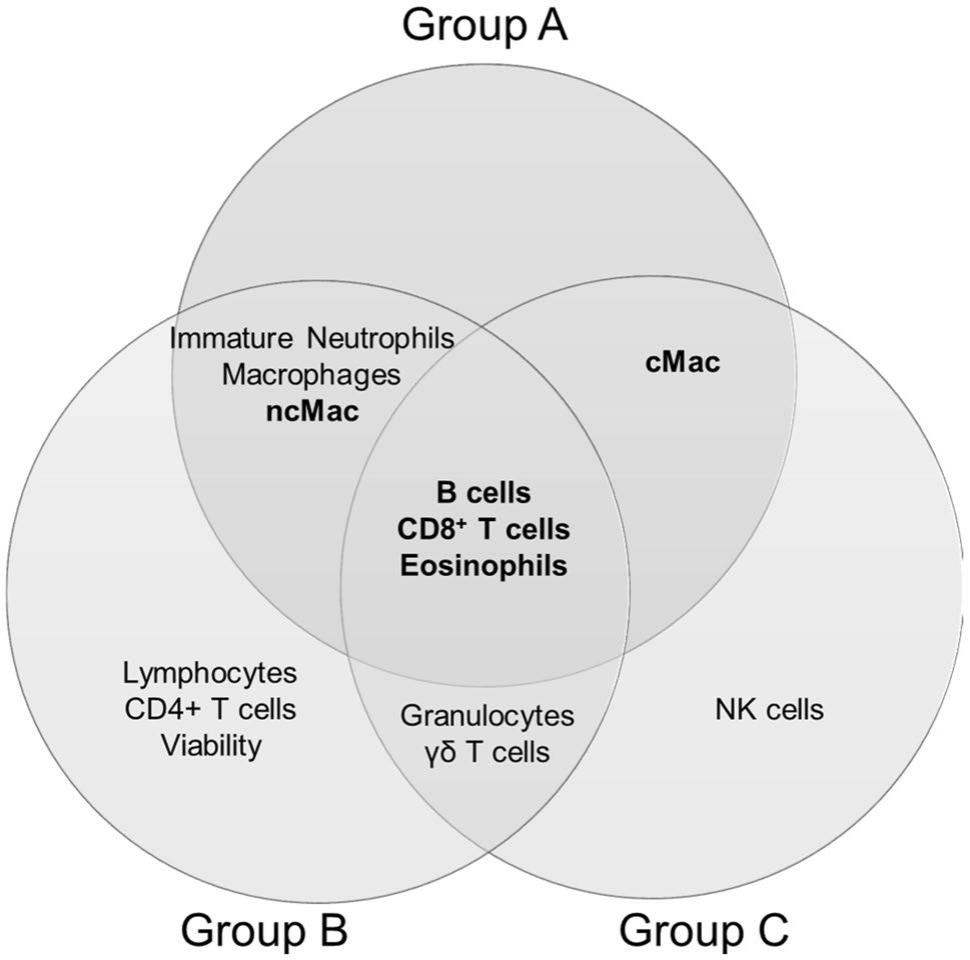
Overview of the most discriminating subpopulations or features of sPLS-DA based on only milk parameter, to separate the treatment phases.

Interestingly, again CD8^+^ T cells, B cells and additionally eosinophils were among the most discriminating factors for all groups in milk (highlighted in Fig. 7). Furthermore, ncMac, the immature neutrophils and the total macrophage population contributed to the differentiation of the phases in groups A and B, cMac in groups A and C and the gamma delta T cells and the total granulocyte population in groups B and C.

Of the external laboratory parameters only the erythrocyte and segmented neutrophil count were among the most discriminating factors for all treatment groups. Haptoglobin as well as GLDH and ß-HBA contributed to the separation of phases of groups B and C. NEFA and the monocyte count on the other hand influenced the separation of the treatment phases for groups A and C.

**Figure 8:**
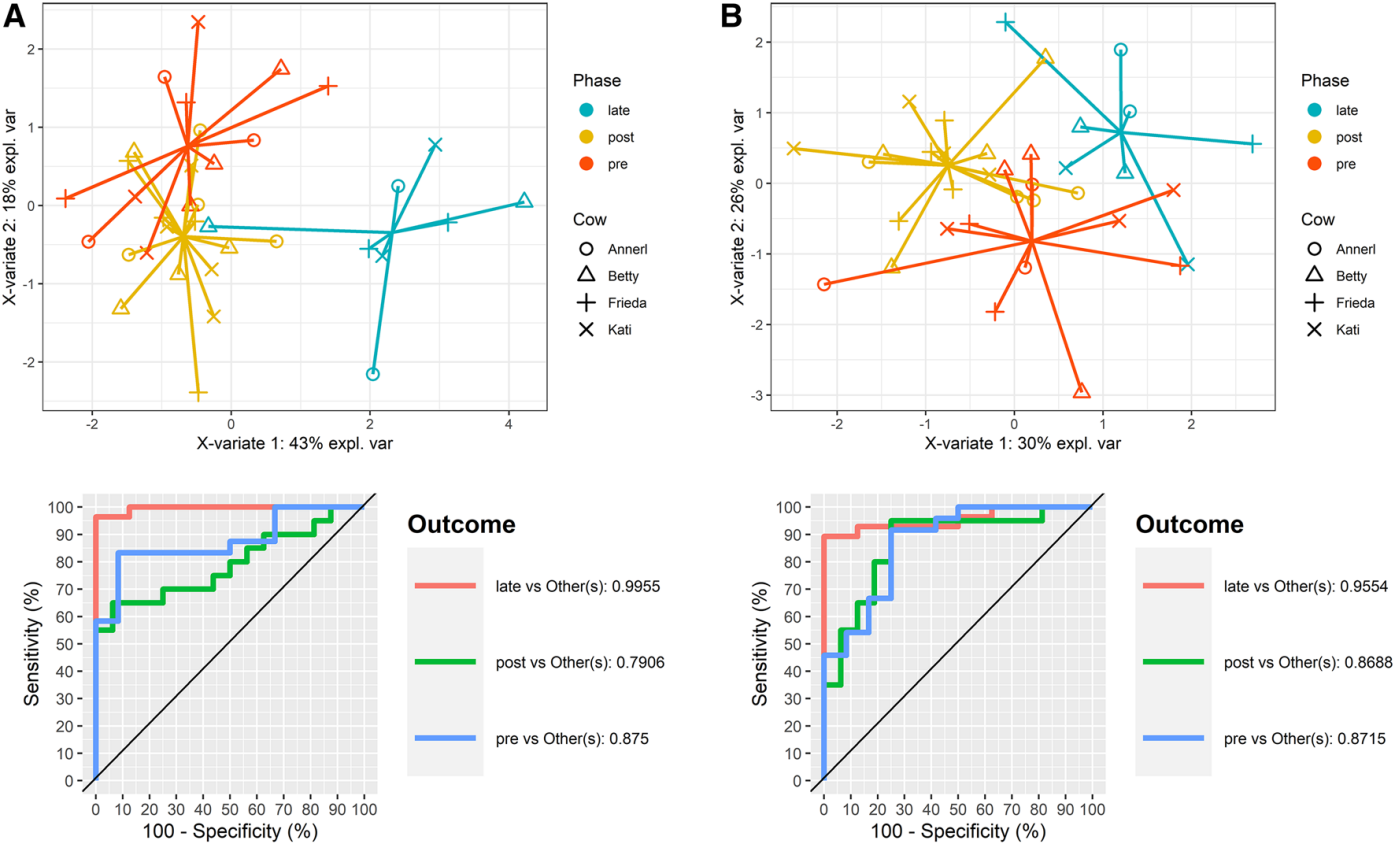
Supervised Clustering of the results of Bovela treatment (group C), three components with only the most promising features. (**A**) PLS-DA using blood parameters (AUROC: “pre” vs others: 0.875, “post” vs others: 0.791, “late” vs others: 0.996); (**B**) PLS-DA using milk parameters (AUROC: “pre” vs others: 0.872, “post” vs others: 0.869, “late” vs others: 0.955).

The most promising features of our HRDCCs for all groups, i.e. CD8^+^ T cells, B cells, eosinophils and the monocyte or macrophage subpopulations respectively, were analyzed in more detail. To verify their validity, a PLS-DA based on only those five features was performed. For these features a clear separation of all phases (pre vs. post vs. late) in group C could be shown and a significant prediction with high validity could be made. The AUROC values were rising up to 0.996 in blood and 0.955 in milk (Fig. 8).

## Discussion

Milk DCC biomarkers have been successfully established solely for the detection and evaluation of intramammary infections^36,37^, but the usefulness of a milk DCC in veterinary diagnostics to detect even systemic immune events has not been studied yet. In a recent study, we could show alterations in blood and milk DCC, in addition to a local T cell response, after immunizations with several vaccines against C. difficile^38^. Since milk sampling is easier and more convenient than taking blood samples, routine milk DCC diagnostics with reliable biomarkers for different diseases could be of great value. We therefore established our immunophenotyping method to detect and quantify cell subpopulations, first in bovine blood, where the isolation and detection of viable cells is quite easy, and second in bovine milk, where comparable cell types and surface markers are present after diapedesis, i.e. the successful migration of almost all blood leukocytes subpopulations through the blood-milk barrier^39^. With this first feasibility study, we showed that our bovine immune screening and the automatic gating is not only methodologically working but also applicable to various immune relevant scenarios, exemplified for vaccinated cows using three different standardized and well-established vaccinations that are based on viral, bacterial or fungal pathogens. By means of our newly developed high-resolution immunophenotyping and the multivariate biostatistical analysis, we recognized measurable and statistical relevant differences in the immune states of these dairy cows, before, during and after vaccinations (see also supplement data for Bovalto Respi 3 and Insol Trichophyton treatments).

### Benefit of an additional automatic gating technique

In general, flow cytometric data is manually analyzed by setting gates to get relative counts of cell subpopulations. The application of an established software tool as FlowJo (Becton, Dickinson and Company) is absolutely necessary to get a good insight into the distribution of cell populations and to find the best way to evaluate these, e.g. by measuring the relative quantities. These complex analysis are very time consuming and bear sources for errors due to subjective gating. During manual analysis, there is always a risk of creating unwanted bias by setting the gates slightly different or inaccurate on various time points^8^, especially if the cell populations are not separated clearly. To avoid this, we were applying an automated gating procedure implemented in R in addition to a manual approach and our results clearly demonstrate its benefits in keeping evaluations reproducible and uniform. Hence, we can recommend this highly reliable automated gating procedure in comparison to time consuming and inexact manual gating, especially in a study that extends over a longer time course or when performing high-resolution immunophenotyping with numerous single evaluations in each biological sample.

### Inconsistencies of our results to the externally analyzed blood cell count

The blood cell counts that were obtained by the external laboratory differed from the results obtained by immunophenotyping. This was possibly caused by the different methods of cell preparation before the analyses. Due to the viability and antibody staining we had to include several washing and centrifugation steps prior to the flow cytometric analysis. Furthermore, we processed the samples shortly after the blood was taken in contrast to the external laboratory, which received the samples only the next day. Considering this, it is necessary for future studies to acquire reference values of healthy cows with our high-resolution screening technique instead of linearly comparing the results to those obtained with a different method.

Especially the monocyte count varies between the two methods. This could have been caused not only by the preparation but could have also been due to the fact, that we identified the monocytes solely based on their SSC/CD45 values (see Fig. 3D2) instead of using their CD172a expression as an additional gating step, as recommended by Grandoni, et al.^40^. For a more reliable identification of monocytes, this should be included in the gating strategy of future studies.

### Outcome of the multivariate analysis

In our multivariate analysis, we first focused on the so far practically well-established standard laboratory parameters to assess their capability to differentiate pre- and post-vaccination phases. Second, we analyzed the data based on our established blood HRDCC to identify the ‘best parameters’ to separate the different treatment phases and different immune statuses before and after vaccinations. Finally, we performed the multivariate analysis based on solely milk HRDCC to disclose the most immune relevant subpopulations in milk, which could serve as new prognostic biomarker.

The current standard to screen the systemic health status of a cow is to analyze blood samples at a veterinary laboratory. For this, a standard differential blood count is performed and key metabolic and inflammatory parameters^41^ are measured, as done in this study. Hence, the separation by sPLS-DA based on the classical standard laboratory parameters was quite good, as expected.

To figure out the advantage of our newly established high-resolution immunophenotyping over the standard laboratory analysis, we performed the clustering methods based on our blood HRDCC. For all treatment groups, CD8^+^ T cells and B cells turned out to be among the most stable and best discriminating markers, herein independent of the applied vaccination. Furthermore, the monocytes subpopulations and the eosinophils played an important role in differentiating the different treatment phases, but the influence was of varying degrees in the individual groups. If this is applicable as well to other immunological settings has to be proven in further future studies or systemic inflammatory scenarios.

Our ultimate goal for the future is to discover early diagnostic biomarker signatures directly in milk, based on the developed high-resolution immunophenotyping. This should definitely not be limited to mastitis or other mammary gland derived diseases, but also be applicable to discover new markers in milk for systemic infections or maladies. These milk biomarkers could help to detect diseases in an early or subclinical state and may eventually be applied later on in a routine testing. In our scenario the influences of all vaccinations were reflected in the milk HRDCC, albeit not always as evident as in the blood HRDCC. This could indicate that other systemic immune responses can be detected via milk HRDCC as well. It was noteworthy that the same cell populations—CD8^+^ T cells, B cells and eosinophils—were among the most discriminating factors and subpopulations for all vaccination types in the sPLS-DA, CD8^+^ T cells and B cells even both in blood and milk. The monocyte/macrophage subpopulations further strengthened the clustering validity of the vaccination phases. Previous studies already suggest that these cell types are involved in the udder immune response. Park et al.^42^ for example showed that cows with a high susceptibility to mastitis had a ratio of CD4^+^ to CD8^+^ T cells of less than one, both in blood and milk. The relation of T cells to B cells can indicate a diseased udder with CD2/CD21 index in milk below ten^10^.

When performing a PLS-DA using only the most promising parameters, i.e. CD8^+^ T cells, B cells, eosinophils and the monocyte or macrophage subpopulations respectively, a good statistical separation was possible in both blood and milk and hence the immune stimulatory effect of all the vaccination could clearly be shown.

Nevertheless, the differentiation of the treatment phases was more pronounced when as much as possible available parameters were considered during the supervised clustering by sPLS-DA. In this case, quantified biomarkers of all measurement layers—including blood derived, milk derived and standard laboratory parameters—seemed to have an influence. It is obvious, that the more variables are added, the better and more significant the separation is. But this is of minor interest for the practical use as the biomarker screening would then have to be performed on multiple layers.

### Role of intermediate monocytes in immune reactions after vaccinations

As the percentage of intermediate monocytes clearly increased after each vaccination with Bovalto Respi 3 and this cell subpopulation also showed to be the major contribution to component 1 in the sPLS-DA of the Bovela treatment group, it seems to be an important factor in the immune reaction to a vaccination. This can be due to their enhanced production of inflammatory cytokines like TNF-α or IL-1β or reactive oxygen species (ROS)^27,43^. High numbers of intM were also found in calves infected with Theileria parva, in which the frequency increased with the progression of a lethal infection^44^. Similar is known for children suffering from acute malaria^45^. In a different approach, skin inflammations were induced in calves with a saponin-based adjuvant—equivalent to the Quillaja saponin adjuvant used in Bovalto Respi 3—that led to a strong recruitment of intM to the draining lymph node^43^.

Since recent studies^46^ suggest that intM and ncM are alike in terms of their transcriptomic profiles and thus also in their functions, the role of the intermediate monocytes in immune responses should be further investigated, for example by using gene expression profiling on sorted monocyte subpopulation after vaccinations.

## Conclusions

This feasibility study with three different vaccination scenarios is the first quantitative application of our new high-resolution immunophenotyping method. Promising early biomarker relevant subpopulations could be identified, first and foremost CD8^+^ T cells and B cells, but also eosinophils, monocyte or macrophage subpopulations, in blood or milk respectively. To identify and establish practical useful and valid biomarker patterns, studies with far more animals of different breeds and milk performance levels are needed, these should include spontaneous occurring diseases, systemic inflammation or immune system stimulating events. In a next approach we will closely monitor dairy cows throughout the entire lactation, i.e. 305 days, to obtain reference values and trace the progression and dynamics of the HRDCC. Interesting subpopulations of immune cells, like CD8^+^ T cells and B cells, could be further examined in more depth and detail, e.g. extracting cells by means of fluorescence-activated cell sorting and investigating the expression of new marker genes by real-time RT-PCR or holistic gene expression studies by next generation sequencing.

## Supplementary Information

Supplementary Information.

## References

[1] Viguier C, Arora S, Gilmartin N, Welbeck K, O'Kennedy R (2009). Mastitis detection: Current trends and future perspectives. Trends Biotechnol..

[2] Schukken YH, Wilson DJ, Welcome F, Garrison-Tikofsky L, Gonzalez RN (2003). Monitoring udder health and milk quality using somatic cell counts. Vet. Res..

[3] Adkins PRF, Middleton JR (2018). Methods for diagnosing mastitis. Vet. Clin. N. Am. Food Anim. Pract..

[4] Sordillo LM (2018). Mammary gland immunobiology and resistance to mastitis. Vet. Clin. N. Am. Food Anim. Pract..

[5] Pilla R, Schwarz D, Konig S, Piccinini R (2012). Microscopic differential cell counting to identify inflammatory reactions in dairy cow quarter milk samples. J. Dairy Sci..

[6] Rivas AL, Quimby FA, Blue J, Coksaygan O (2001). Longitudinal evaluation of bovine mammary gland health status by somatic cell counting, flow cytometry, and cytology. J. Vet. Diagn. Invest..

[7] Schwarz D (2011). Flow cytometric differential cell counts in milk for the evaluation of inflammatory reactions in clinically healthy and subclinically infected bovine mammary glands. J. Dairy Sci..

[8] Cossarizza A (2017). Guidelines for the use of flow cytometry and cell sorting in immunological studies. Eur. J. Immunol..

[9] Pilla R (2013). Differential cell count as an alternative method to diagnose dairy cow mastitis. J. Dairy Sci..

[10] Schwarz D (2013). CD2/CD21 index: A new marker to evaluate udder health in dairy cows. J. Dairy Sci..

[11] Degen S (2015). Cell differentiation assisting in evaluating mastitis treatment prognosis. Milchwissenschaft.

[12] Holm, C. Method for determining a degree of infection. EP2630487 (2012).

[13] Damm M, Holm C, Blaabjerg M, Bro MN, Schwarz D (2017). Differential somatic cell count-A novel method for routine mastitis screening in the frame of Dairy Herd Improvement testing programs. J. Dairy Sci..

[14] Kand D, Castro-Montoya J, Selje-Assmann N, Dickhoefer U (2021). The effects of rumen nitrogen balance on intake, nutrient digestibility, chewing activity, and milk yield and composition in dairy cows vary with dietary protein sources. J. Dairy Sci..

[15] Roth FX, Schwarz FJ, Stangl GI (2011). Kirchgessner Tierernährung.

[16] Brodersen BW (2010). Bovine respiratory syncytial virus. Vet. Clin. N. Am. Food Anim. Pract..

[17] Confer AW, Ayalew S (2018). Mannheimia haemolytica in bovine respiratory disease: Immunogens, potential immunogens, and vaccines. Anim. Health Res. Rev..

[18] Schaufuss P, Steller U (2004). Immunological responses to dermatophyte infections: A literature review. Tieraerztl Umschau.

[19] Smith JM, Griffin JF (1995). Strategies for the development of a vaccine against ringworm. J. Med. Vet. Mycol..

[20] Meyers G (2007). Bovine viral diarrhea virus: prevention of persistent fetal infection by a combination of two mutations affecting Erns RNase and Npro protease. J. Virol..

[21] Platt R, Kesl L, Guidarini C, Wang C, Roth JA (2017). Comparison of humoral and T-cell-mediated immune responses to a single dose of Bovela((R)) live double deleted BVDV vaccine or to a field BVDV strain. Vet. Immunol. Immunopathol..

[22] Koess C, Hamann J (2008). Detection of mastitis in the bovine mammary gland by flow cytometry at early stages. J. Dairy Res..

[23] Loken MR, Brosnan JM, Bach BA, Ault KA (1990). Establishing optimal lymphocyte gates for immunophenotyping by flow cytometry. Cytometry.

[24] Weil GJ, Chused TM (1981). Eosinophil autofluorescence and its use in isolation and analysis of human eosinophils using flow microfluorometry. Blood.

[25] Mayeno AN, Hamann KJ, Gleich GJ (1992). Granule-associated flavin adenine dinucleotide (FAD) is responsible for eosinophil autofluorescence. J. Leukoc. Biol..

[26] Van Merris V, Meyer E, Burvenich C (2002). Functional maturation during bovine granulopoiesis. J. Dairy Sci..

[27] Hussen J (2013). Phenotypic and functional heterogeneity of bovine blood monocytes. PLoS ONE.

[28] Storset AK (2004). NKp46 defines a subset of bovine leukocytes with natural killer cell characteristics. Eur. J. Immunol..

[29] Naessens J (1990). Characterization of a bovine leucocyte differentiation antigen of 145,000 MW restricted to B lymphocytes. Immunology.

[30] Finak G (2014). OpenCyto: an open source infrastructure for scalable, robust, reproducible, and automated, end-to-end flow cytometry data analysis. PLoS Comput. Biol..

[31] Finak, G. & Jiang, M. flowWorkspace: Infrastructure for representing and interacting with gated and ungated cytometry data sets. R package version 3.33.7. (2019).

[32] Alsemgeest SP (1994). Concentrations of serum amyloid-A (SAA) and haptoglobin (HP) as parameters of inflammatory diseases in cattle. Vet. Q.

[33] R: A language and environment for statistical computing. R Foundation for Statistical Computing, Vienna, Austria (2020).

[34] factoextra: Extract and Visualize the Results of Multivariate Data Analyses. R package version 1.0.7. (2020).

[35] Rohart F, Gautier B, Singh A, Le Cao KA (2017). mixOmics: An R package for 'omics feature selection and multiple data integration. PLoS Comput. Biol..

[36] Goncalves JL (2017). Using milk leukocyte differentials for diagnosis of subclinical bovine mastitis. J. Dairy Res..

[37] Kirkeby C (2020). Differential somatic cell count as an additional indicator for intramammary infections in dairy cows. J. Dairy Sci..

[38] Schmautz C (2018). Immune cell counts and signaling in body fluids of cows vaccinated against Clostridium difficile. J. Biol. Res..

[39] Sordillo LM, Streicher KL (2002). Mammary gland immunity and mastitis susceptibility. J. Mammary Gland Biol. Neoplasia.

[40] Grandoni F (2021). Comprehensive phenotyping of peripheral blood monocytes in healthy bovine. Cytometry A.

[41] Petersen HH, Nielsen JP, Heegaard PM (2004). Application of acute phase protein measurements in veterinary clinical chemistry. Vet. Res..

[42] Park YH (2004). Characterization of lymphocyte subpopulations and major histocompatibility complex haplotypes of mastitis-resistant and susceptible cows. J. Vet. Sci..

[43] Lund H, Boysen P, Akesson CP, Lewandowska-Sabat AM, Storset AK (2016). Transient migration of large numbers of CD14(++) CD16(+) monocytes to the draining lymph node after onset of inflammation. Front. Immunol..

[44] Bastos RG, Sears K, Dinkel KD, Knowles DP, Fry LM (2019). Changes in the molecular and functional phenotype of bovine monocytes during Theileria parva infection. Infect. Immun..

[45] Dobbs KR (2017). Monocyte dysregulation and systemic inflammation during pediatric falciparum malaria. JCI Insight.

[46] Talker SC (2018). Precise delineation and transcriptional characterization of bovine blood dendritic-cell and monocyte subsets. Front. Immunol..

