# Supplementary material for: Development of an advanced flow cytometry based high-resolution immunophenotyping method to benchmark early immune response in dairy cows
Source: Sci Rep. 2021 Nov 24;11:22896. doi: 10.1038/s41598-021-02405-w (PMC8613216; doi:10.1038/s41598-021-02405-w)
Supplement: Supplementary file 1 — Supplementary Information. [file 41598_2021_2405_MOESM1_ESM.pdf]

Supplementary File

Development of an advanced flow cytometry based high-resolution immunophenotyping method to benchmark early immune response in dairy cows

S. Farschtschi, M. Mattes, A. Hildebrandt, D. Chiang, B. Kirchner, H. Kliem and M. W. Pfaffl

Supplementary Table S1. Composition of the six used mastermixes.

Mastermix 1 for Live/Dead Staining, reagent in DPBS	
Zombie NIR Fixable Viability Kit (Biolegend, Inc.)	1:800
Mastermix 2 with primary antibodies for 5 color run, reagents diluted in FACS buffer	
Mouse anti-sheep CD45, Biotin, IgG1, clone 1.11.32 (BioRad Laboratories Inc.)	1:250
Mouse anti-bovine CD11b, unlabeled, IgG2b, clone MM10A (Monoclonal Antibody Center, Washington State University, USA)	1:400
Mouse anti-human CD14, BV711, IgG2a, clone M5E2 (Becton, Dickinson and Company)	1:66
Mouse anti-human CD16, FITC, IgG2a, clone KD1 (BioRad Laboratories Inc.)	1:166
Mastermix 3 with primary antibodies for 6 color run, reagents diluted in FACS buffer	
Mouse anti-sheep CD45, Biotin, IgG1, clone 1.11.32 (BioRad Laboratories Inc.)	1:250
Mouse anti-bovine CD11b, unlabeled, IgG2b, clone MM10A (Monoclonal Antibody Center, Washington State University, USA)	1:400

Mouse anti-human CD14, BV711, IgG2a, clone M5E2 (Becton, Dickinson and Company)	1:66
Mouse anti-human CD16, FITC, IgG2a, clone KD1 (BioRad Laboratories Inc.)	1:166
Cytokeratin pan monoclonal antibody, APC, IgG1, clone C-11 (Thermo Fisher Scientific Inc.)	1:400
Mastermix 4 with primary antibodies for 7 color run, reagents diluted in FACS buffer	
Mouse anti-sheep CD45, Biotin, IgG1, clone 1.11.32 (BioRad Laboratories Inc.)	1:250
Mouse anti-bovine CD4, Alexa Fluor 647, IgG2a, clone CC8 (BioRad Laboratories Inc.)	1:250
Mouse anti-bovine CD8, unlabeled, IgG3, clone CACT130A (Monoclonal Antibody Center, Washington State University, USA)	1:250
Mouse anti-bovine CD21, PE-Cy7, IgG1, clone LT21 (EXBIO Praha, a.s.)	1:200
Mouse anti-bovine CD335, PE, IgG1, clone AKS1 (BioRad Laboratories Inc.)	1:20
Mouse anti-bovine γδ TCR1-N24 δ chain, IgG2b, clone GB21A (Monoclonal Antibody Center, Washington State University, USA)	1:250
Mastermix 5 with secondary antibodies for 5 and 6 color run, reagents diluted in FACS buffer	
Streptavidin BV786 (Becton, Dickinson and Company)	1:800
Rat anti-mouse IgG2b, BV605, clone R12-3 (Becton, Dickinson and Company)	1:1,000

Mastermix 6 with secondary antibodies for 7 color run, reagents diluted in FACS buffer	
Streptavidin BV786 (Becton, Dickinson and Company)	1:800
Rat anti-mouse IgG3, BV421, clone R40-82 (Becton, Dickinson and Company)	1:400
Rat anti-mouse IgG2b, FITC, clone m2b-25G4 (Thermo Fisher Scientific Inc.)	1:1,000

Supplementary Table S2. Concentration of the isotype antibodies used for FMO controls.

Isotype Control Antibodies	
Mouse IgG2a, Alexa Fluor 647 (BioRad Laboratories Inc.)	1:250
Mouse IgG2a, BV711 (Becton, Dickinson and Company)	1:66
Mouse IgG2a, FITC (BioRad Laboratories Inc.)	1:166
Mouse IgG1, PE-Cy7 (EXBIO Praha, a.s.)	1:200
Mouse IgG1, PE (BioRad Laboratories Inc.)	1:20
Mouse IgG1, APC (Thermo Fisher Scientific Inc.)	1:800

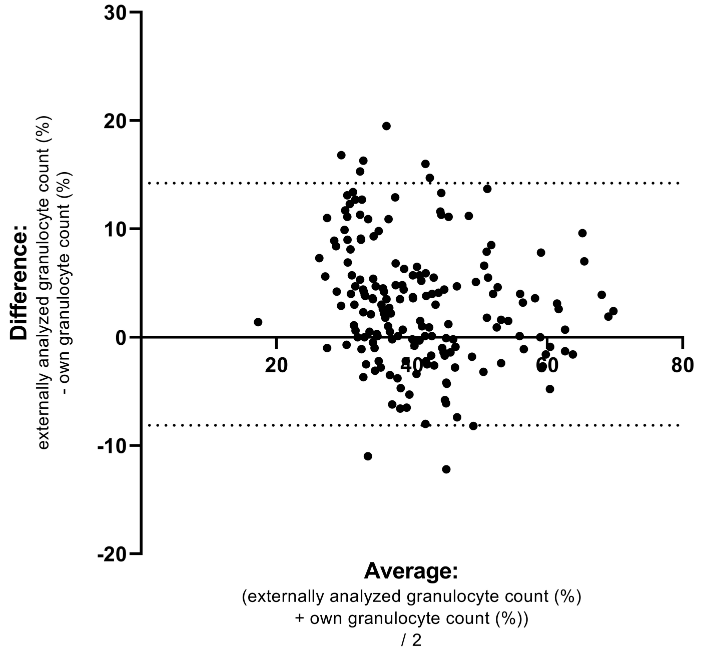

Supplementary Figure S1. Bland-Altman plot comparing the results of the externally analyzed granulocyte count to own results (bias: 3.041, SD of bias: 5.707, 95% limits of agreement from -8.144 to 14.23).

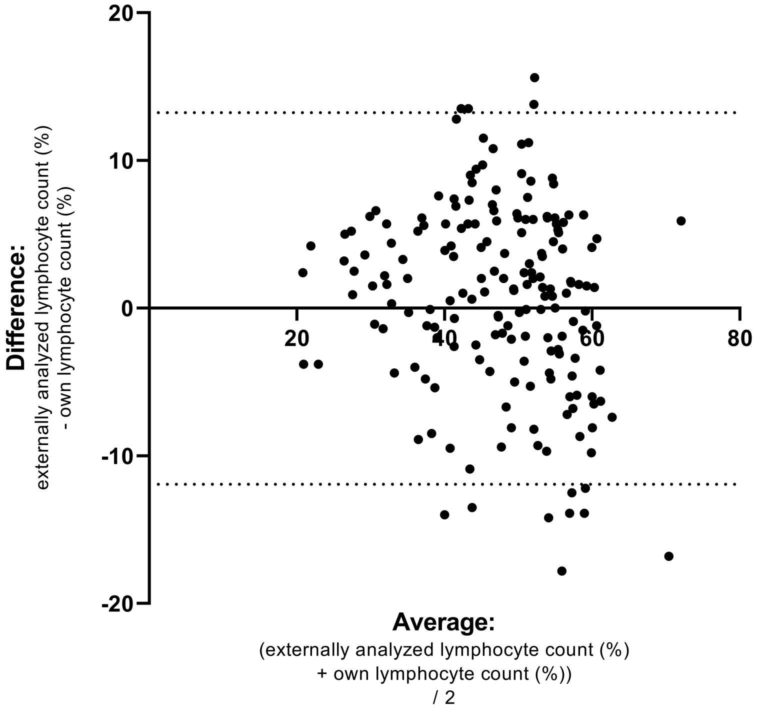

Supplementary Figure S2. Bland-Altman plot comparing the results of the externally analyzed lymphocyte count to own results (bias: 0.6519, SD of bias: 6.420, 95% limits of agreement from -11.93 to 13.23).

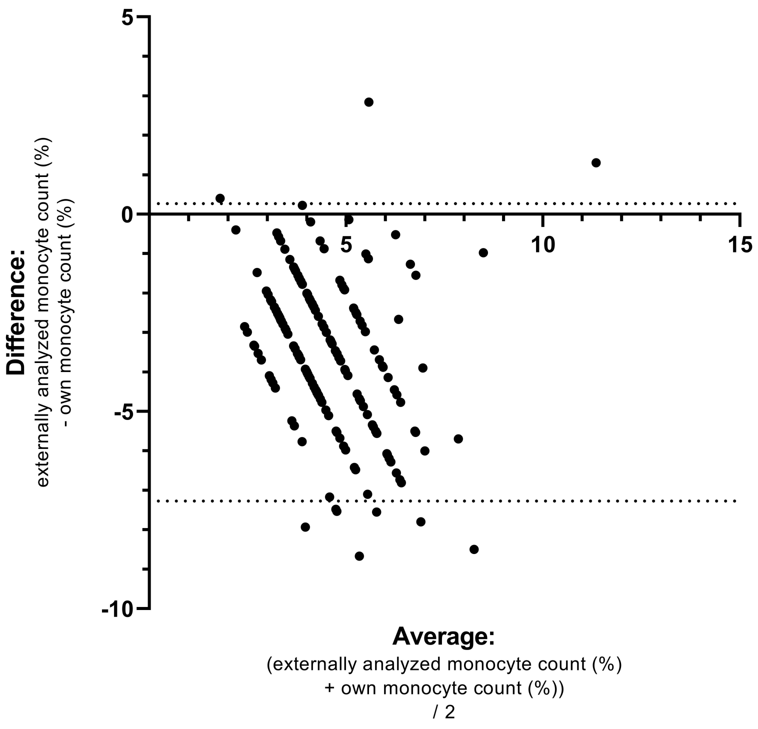

Supplementary Figure S3. Bland-Altman plot comparing the results of the externally analyzed monocyte count to own results (bias: -3.503, SD of bias: 1.922, 95% limits of agreement from -7.270 to 0.2651).

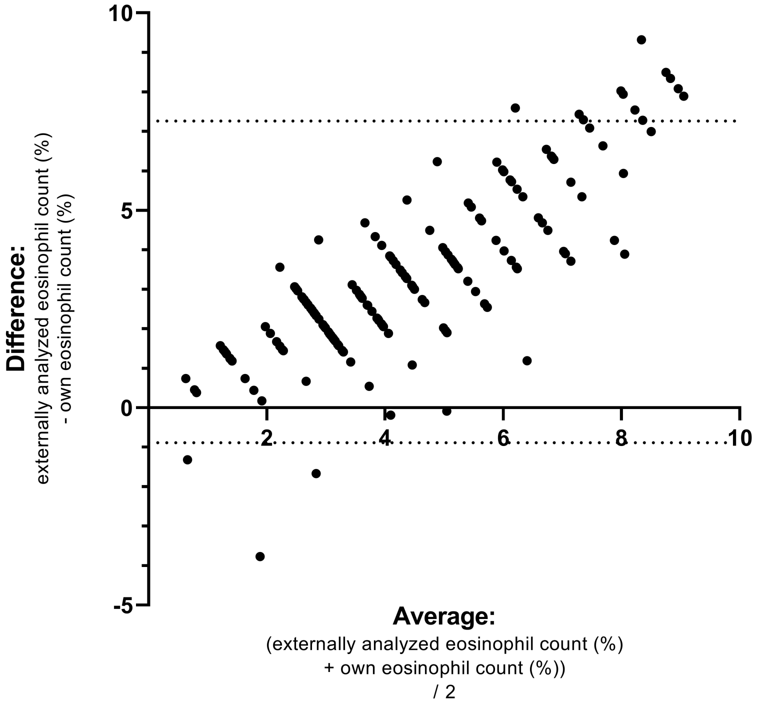

Supplementary Figure S4. Bland-Altman plot comparing the results of the externally analyzed eosinophil count to own results (bias: 3.188, SD of bias: 2.079, 95% limits of agreement from -0.8867 to 7.263).

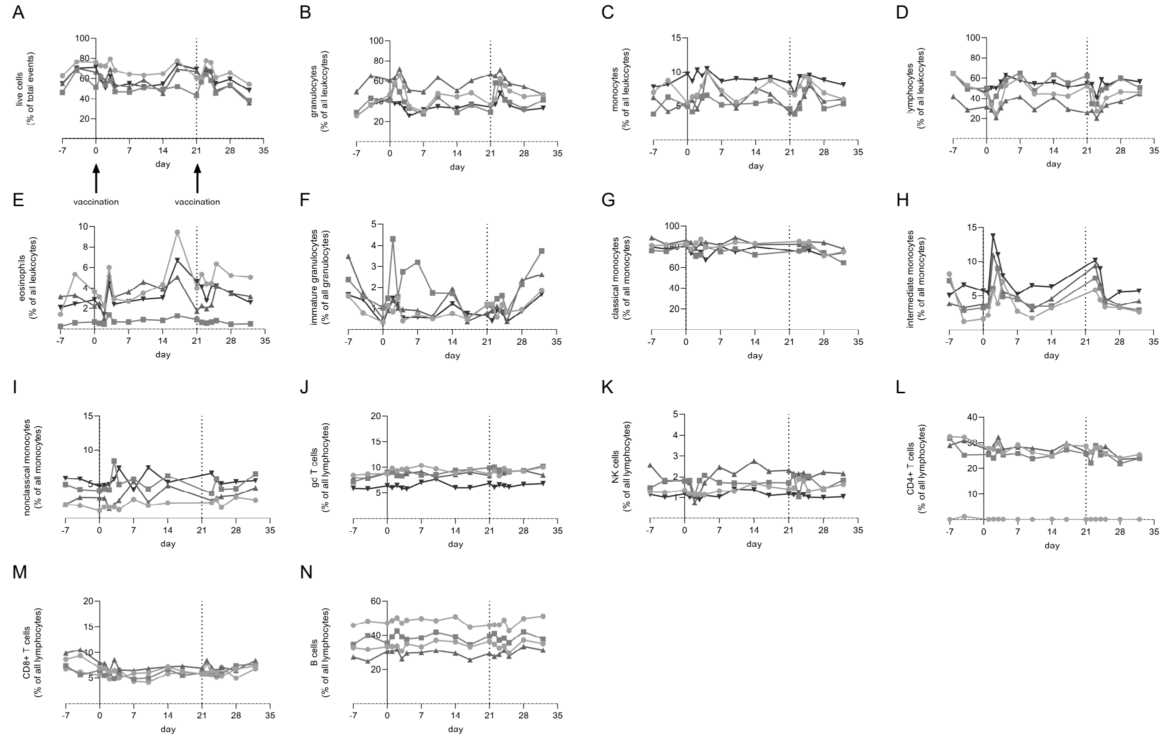

Supplementary Figure S5. Percentages of cell populations in blood, Bovalto Respi 3 treatment (group A). A: live cells as percentage of total events; B: granulocytes as percentage of all leukocytes; C: monocytes as percentage of all leukocytes; D: lymphocytes as percentage of all leukocytes; E: eosinophils as percentage of all leukocytes; F: immature granulocytes as percentage of all granulocytes; G: classical monocytes as percentage of all monocytes; H: intermediate monocytes as percentage of all monocytes; I: nonclassical monocytes as percentage of all monocytes; J: gamma delta T cells as percentage of all lymphocytes; K: natural killer cells as percentage of all lymphocytes; L: $CD4^+$ T cells as percentage of all lymphocytes; M: $CD8^+$ T cells as percentage of all lymphocytes; N: B cells as percentage of all lymphocytes.

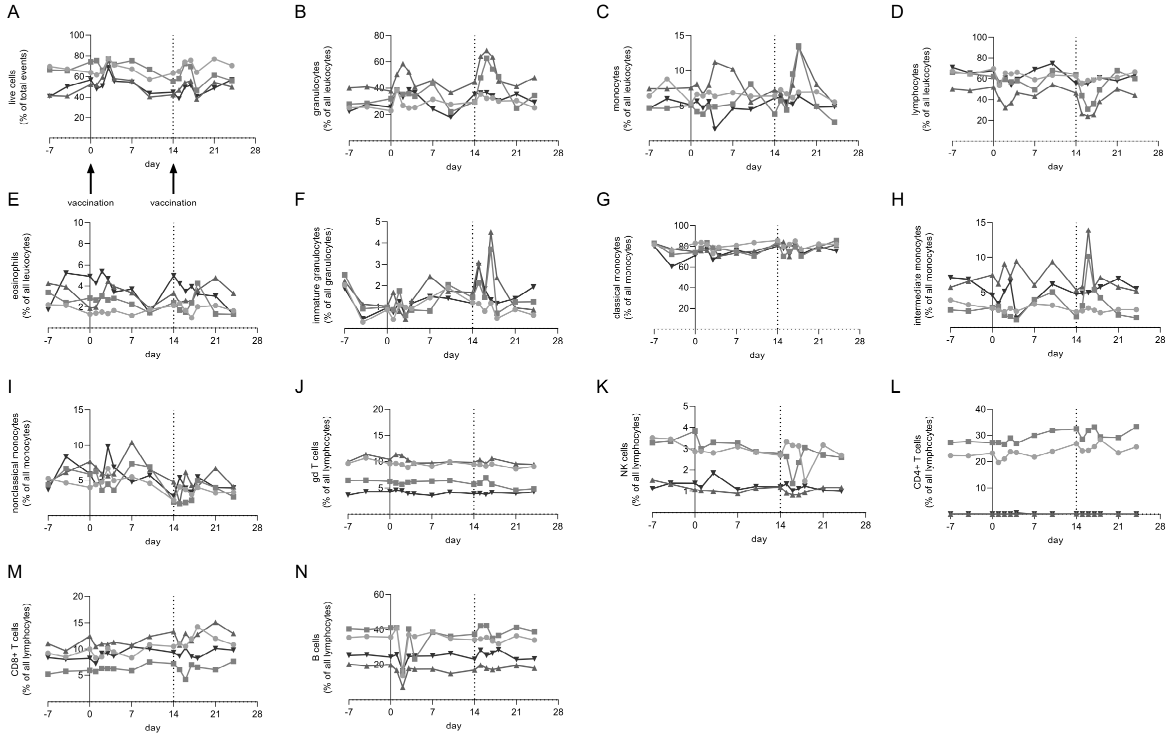

Supplementary Figure S6. Percentages of cell populations in blood, Insol Trichophyton treatment (group B). A: live cells as percentage of total events; B: granulocytes as percentage of all leukocytes; C: monocytes as percentage of all leukocytes; D: lymphocytes as percentage of all leukocytes; E: eosinophils as percentage of all leukocytes; F: immature granulocytes as percentage of all granulocytes; G: classical monocytes as percentage of all monocytes; H: intermediate monocytes as percentage of all monocytes; I: nonclassical monocytes as percentage of all monocytes; J: gamma delta T cells as percentage of all lymphocytes; K: natural killer cells as percentage of all lymphocytes; L: CD4$^+$ T cells as percentage of all lymphocytes; M: CD8$^+$ T cells as percentage of all lymphocytes; N: B cells as percentage of all lymphocytes.

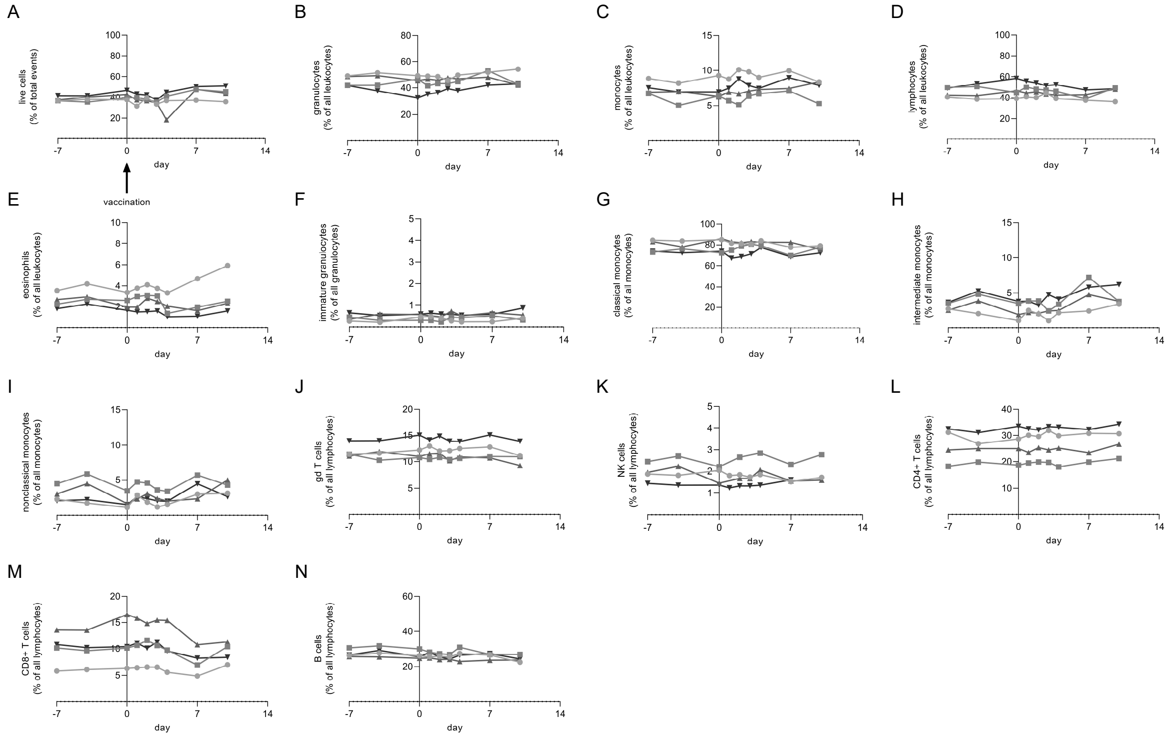

Supplementary Figure S7. Percentages of cell populations in blood, Bovela treatment (group C). A: live cells as percentage of total events; B: granulocytes as percentage of all leukocytes; C: monocytes as percentage of all leukocytes; D: lymphocytes as percentage of all leukocytes; E: eosinophils as percentage of all leukocytes; F: immature granulocytes as percentage of all granulocytes; G: classical monocytes as percentage of all monocytes; H: intermediate monocytes as percentage of all monocytes; I: nonclassical monocytes as percentage of all monocytes; J: gamma delta T cells as percentage of all lymphocytes; K: natural killer cells as percentage of all lymphocytes; L: $CD4^+$ T cells as percentage of all lymphocytes; M: $CD8^+$ T cells as percentage of all lymphocytes; N: B cells as percentage of all lymphocytes.

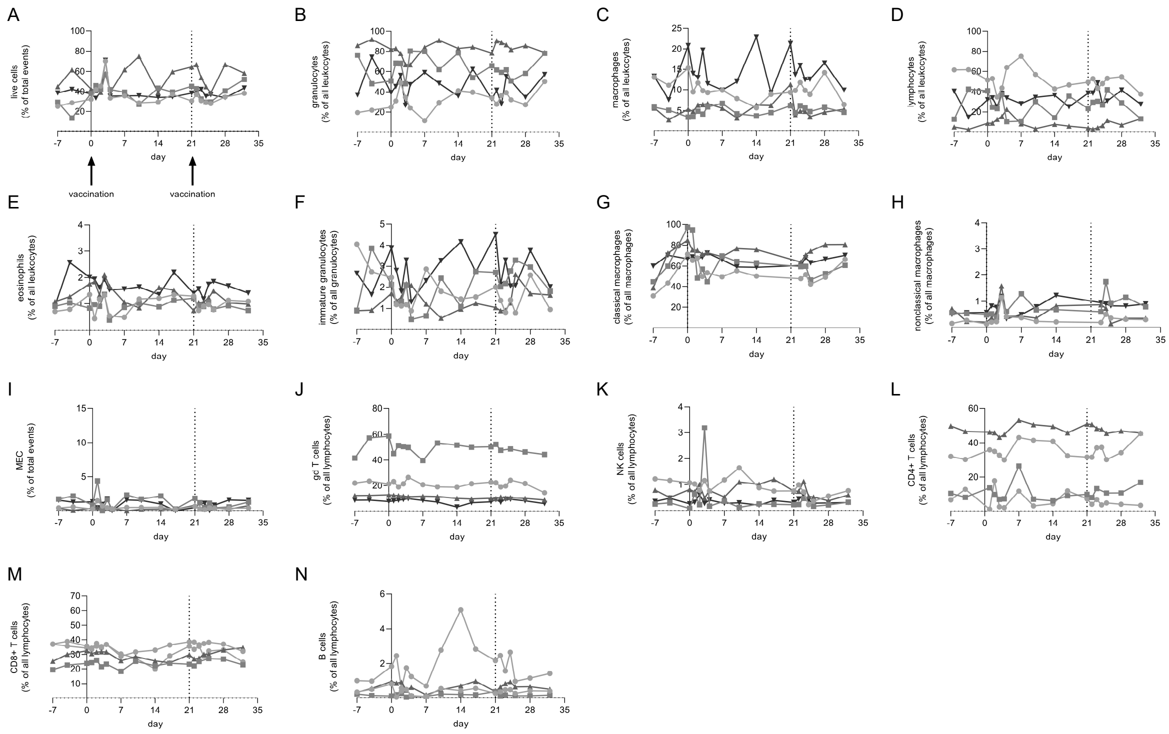

Supplementary Figure S8. Percentages of cell populations in milk, Bovalto Respi 3 treatment (group A). A: live cells as percentage of total events; B: granulocytes as percentage of all leukocytes; C: macrophages as percentage of all leukocytes; D: lymphocytes as percentage of all leukocytes; E: eosinophils as percentage of all leukocytes; F: immature granulocytes as percentage of all granulocytes; G: classical macrophages as percentage of all macrophages; H: nonclassical macrophages as percentage of all macrophages; I: mammary gland epithelial cells as percentage of total events; J: gamma delta T cells as percentage of all lymphocytes; K: natural killer cells as percentage of all lymphocytes; L: $CD4^+$ T cells as percentage of all lymphocytes; M: $CD8^+$ T cells as percentage of all lymphocytes; N: B cells as percentage of all lymphocytes.

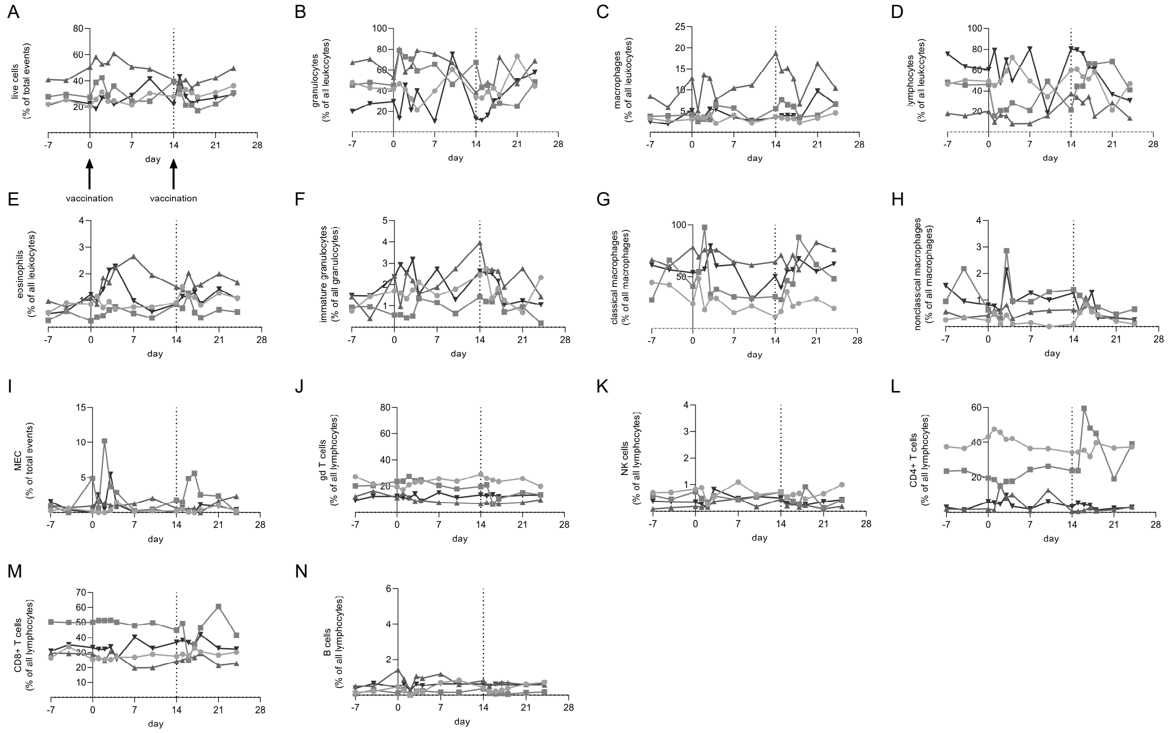

Supplementary Figure S9. Percentages of cell populations in milk, Insol Trichophyton treatment (group B). A: live cells as percentage of total events; B: granulocytes as percentage of all leukocytes; C: macrophages as percentage of all leukocytes; D: lymphocytes as percentage of all leukocytes; E: eosinophils as percentage of all leukocytes; F: immature granulocytes as percentage of all granulocytes; G: classical macrophages as percentage of all macrophages; H: nonclassical macrophages as percentage of all macrophages; I: mammary gland epithelial cells as percentage of total events; J: gamma delta T cells as percentage of all lymphocytes; K: natural killer cells as percentage of all lymphocytes; L: $CD4^+$ T cells as percentage of all lymphocytes; M: $CD8^+$ T cells as percentage of all lymphocytes; N: B cells as percentage of all lymphocytes.

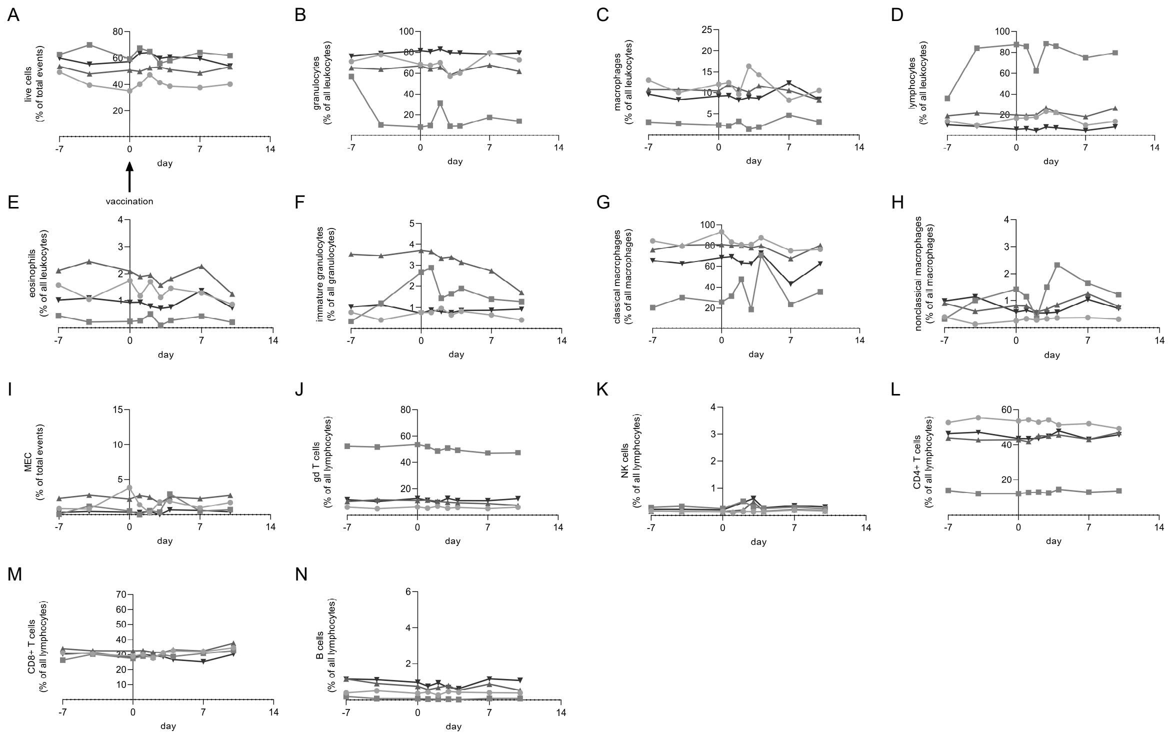

Supplementary Figure S10. Percentages of cell populations in milk, Bovela treatment (group C). A: live cells as percentage of total events; B: granulocytes as percentage of all leukocytes; C: macrophages as percentage of all leukocytes; D: lymphocytes as percentage of all leukocytes; E: eosinophils as percentage of all leukocytes; F: immature granulocytes as percentage of all granulocytes; G: classical macrophages as percentage of all macrophages; H: nonclassical macrophages as percentage of all macrophages; I: mammary gland epithelial cells as percentage of total events; J: gamma delta T cells as percentage of all lymphocytes; K: natural killer cells as percentage of all lymphocytes; L: $CD4^+$ T cells as percentage of all lymphocytes; M: $CD8^+$ T cells as percentage of all lymphocytes; N: B cells as percentage of all lymphocytes.

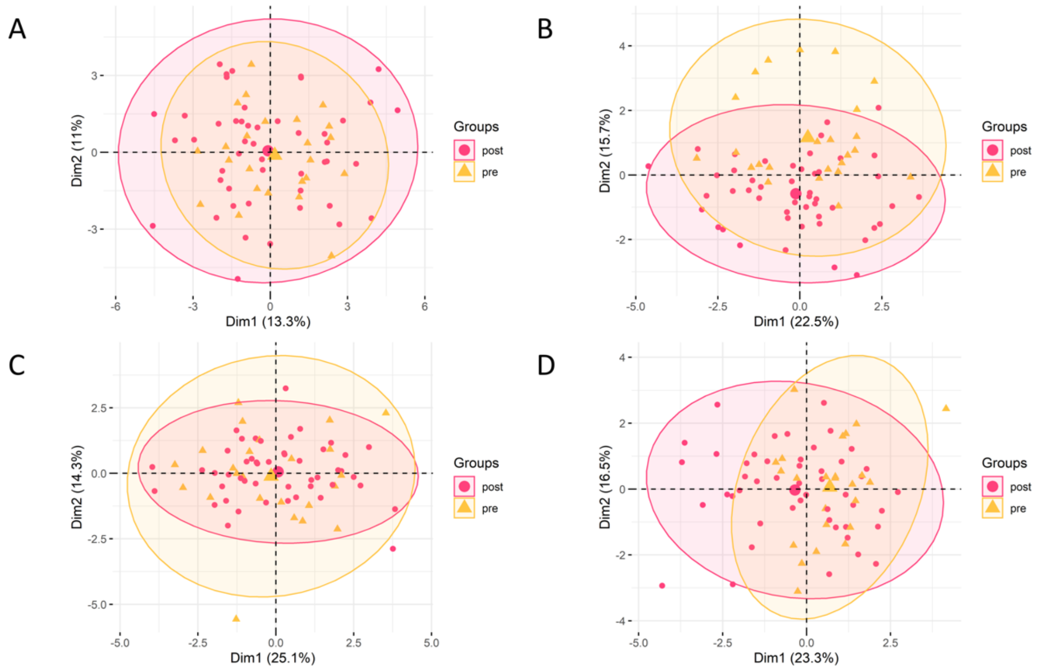

Supplementary Figure S11. Unsupervised clustering of the results of Bovalto Respi 3 treatment (group A). A: PCA using all parameters; B: PCA using blood parameters; C: PCA using milk parameters; D: PCA using parameters analyzed by external laboratory.

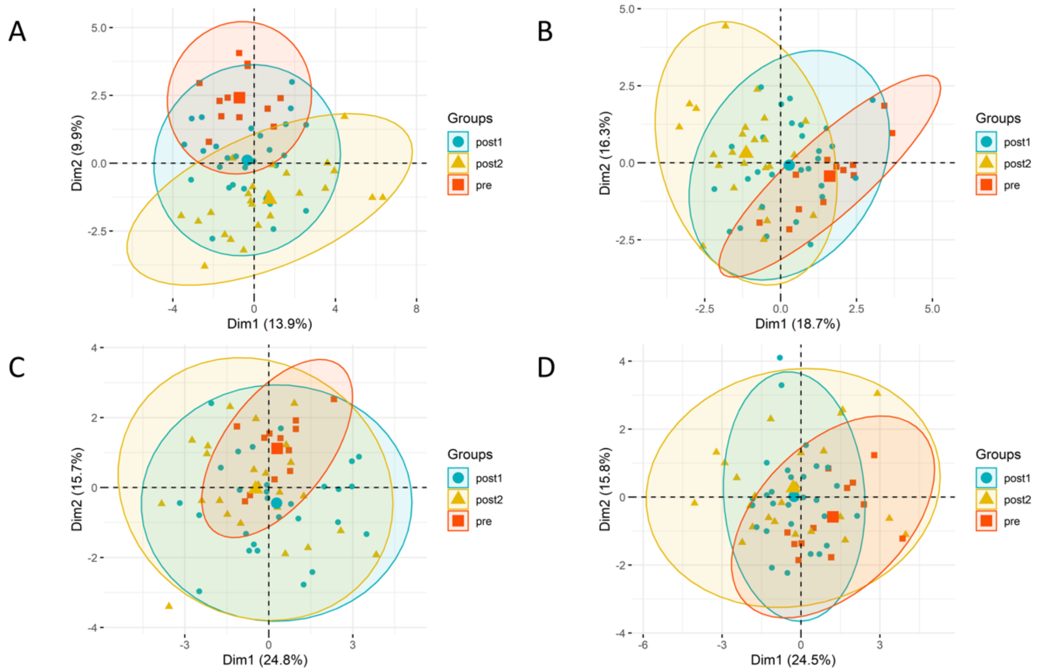

Supplementary Figure S12. Unsupervised clustering of the results of Insol Trichophyton treatment (group B). A: PCA using all parameters; B: PCA using blood parameters; C: PCA using milk parameters; D: PCA using parameters analyzed by external laboratory.

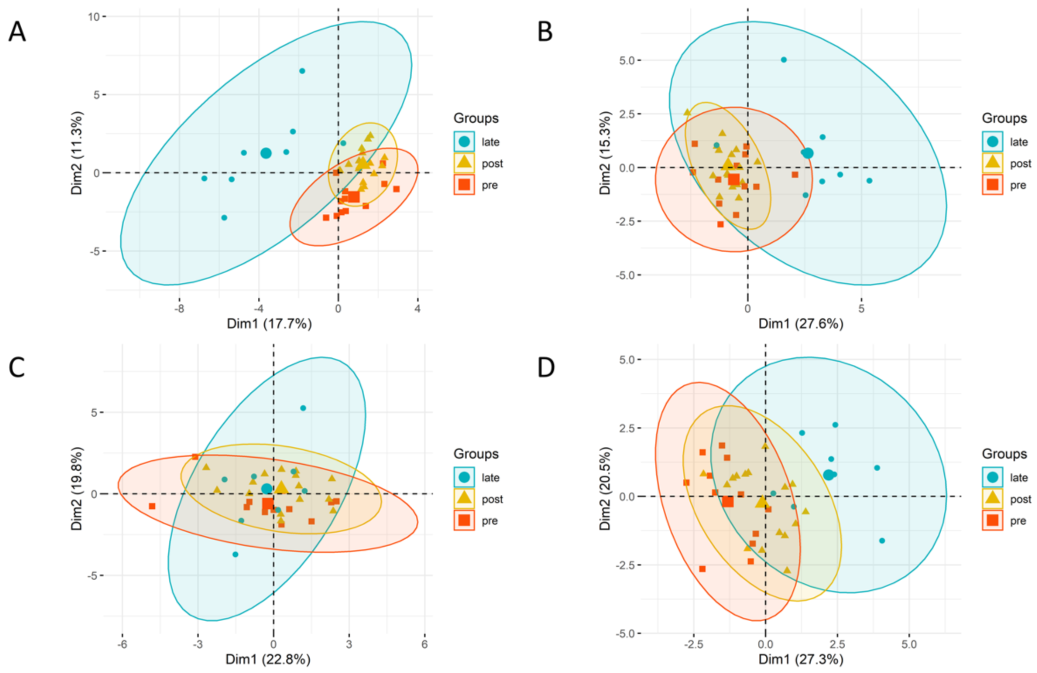

Supplementary Figure S13. Unsupervised clustering of the results of Bovela treatment (group C). A: PCA using all parameters; B: PCA using blood parameters; C: PCA using milk parameters; D: PCA using parameters analyzed by external laboratory.

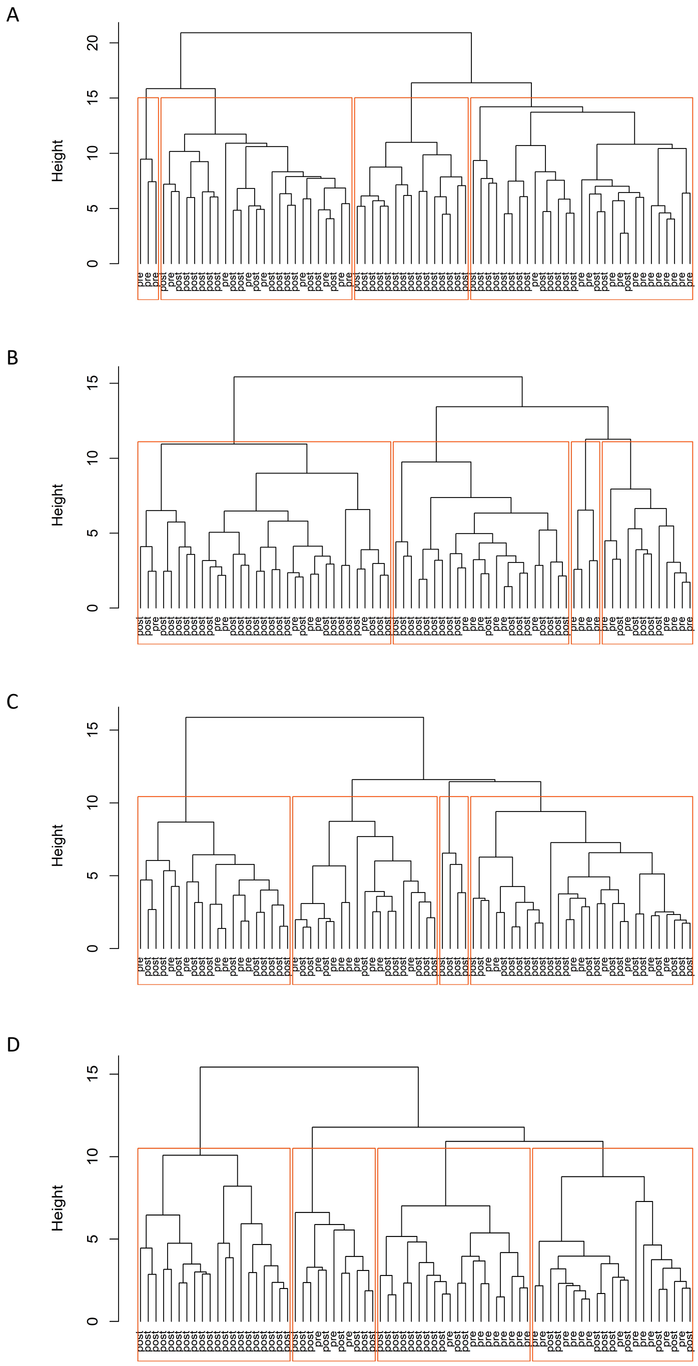

Supplementary Figure S14. Unsupervised hierarchical clustering of the results of Bovalto Respi 3 treatment (group A). A: HC using all parameters; B: HC using blood parameters; C: HC using milk parameters; D: HC using parameters analyzed by external laboratory.

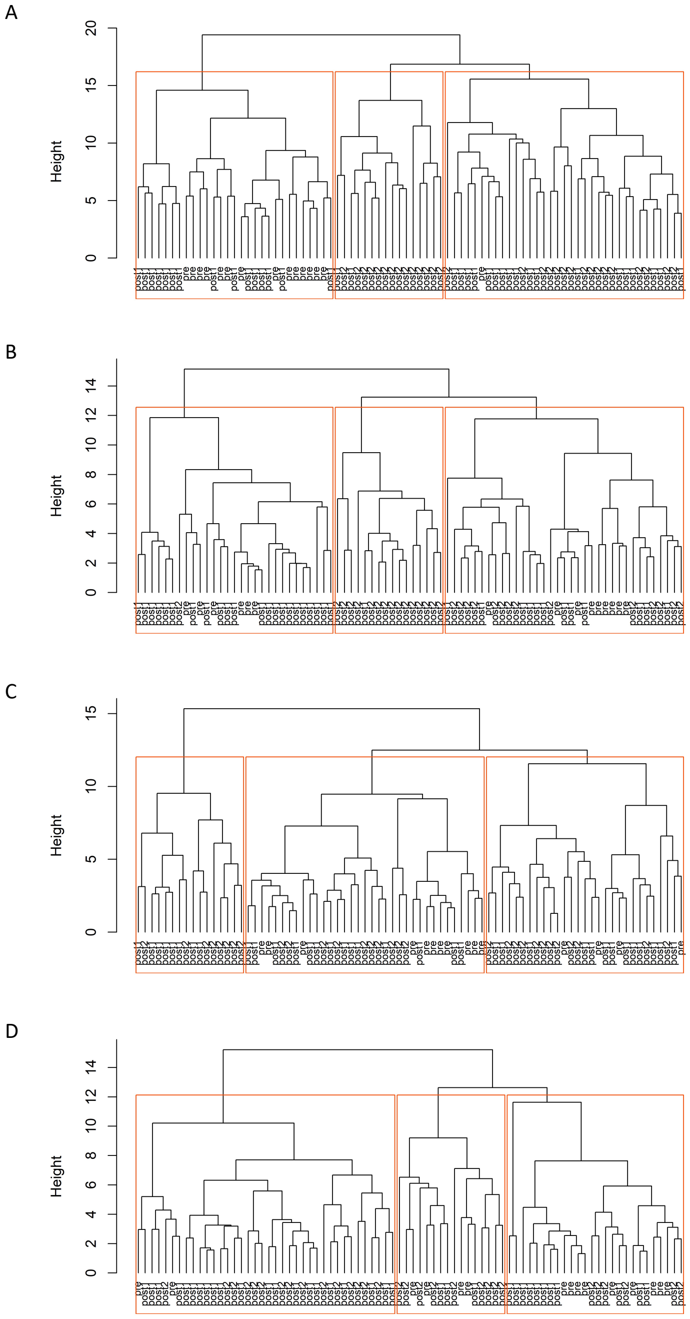

Supplementary Figure S15. Unsupervised hierarchical clustering of the results of Insol Trichophyton treatment (group B). A: HC using all parameters; B: HC using blood parameters; C: HC using milk parameters; D: HC using parameters analyzed by external laboratory.

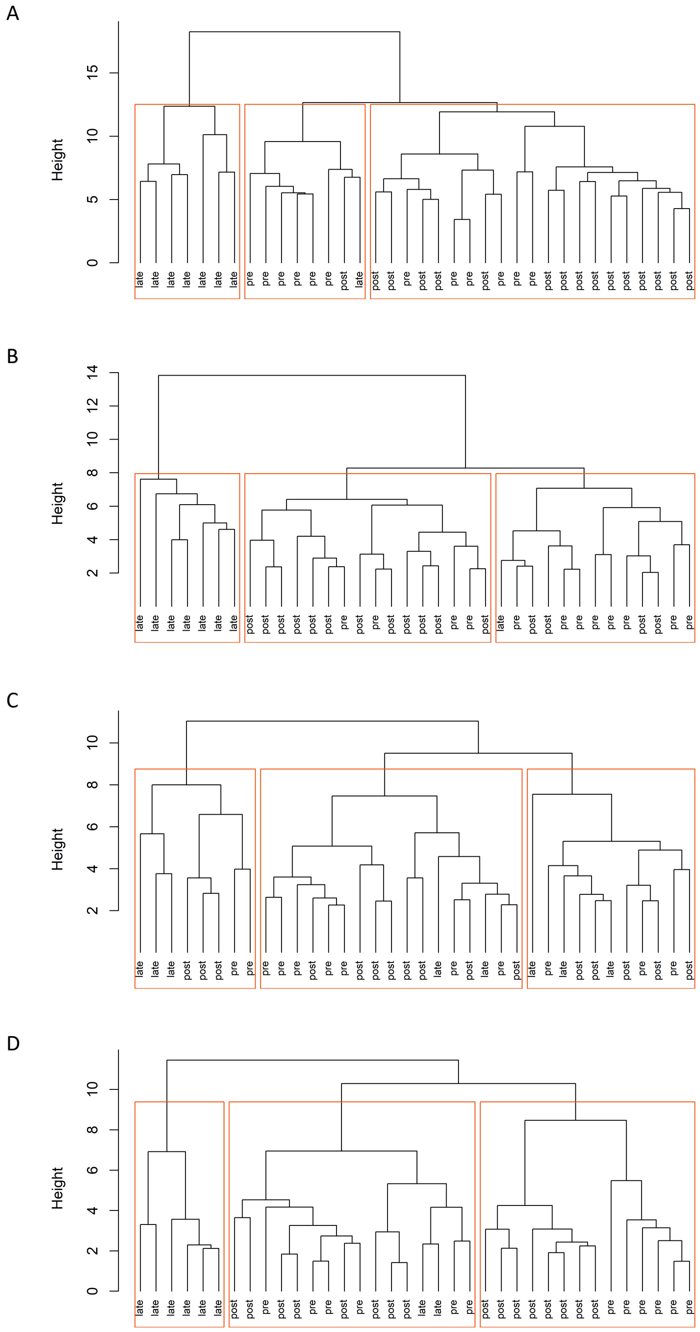

Supplementary Figure S16. Unsupervised hierarchical clustering of the results of Bovela (group C). A: HC using all parameters; B: HC using blood parameters; C: HC using milk parameters; D: HC using parameters analyzed by external laboratory.

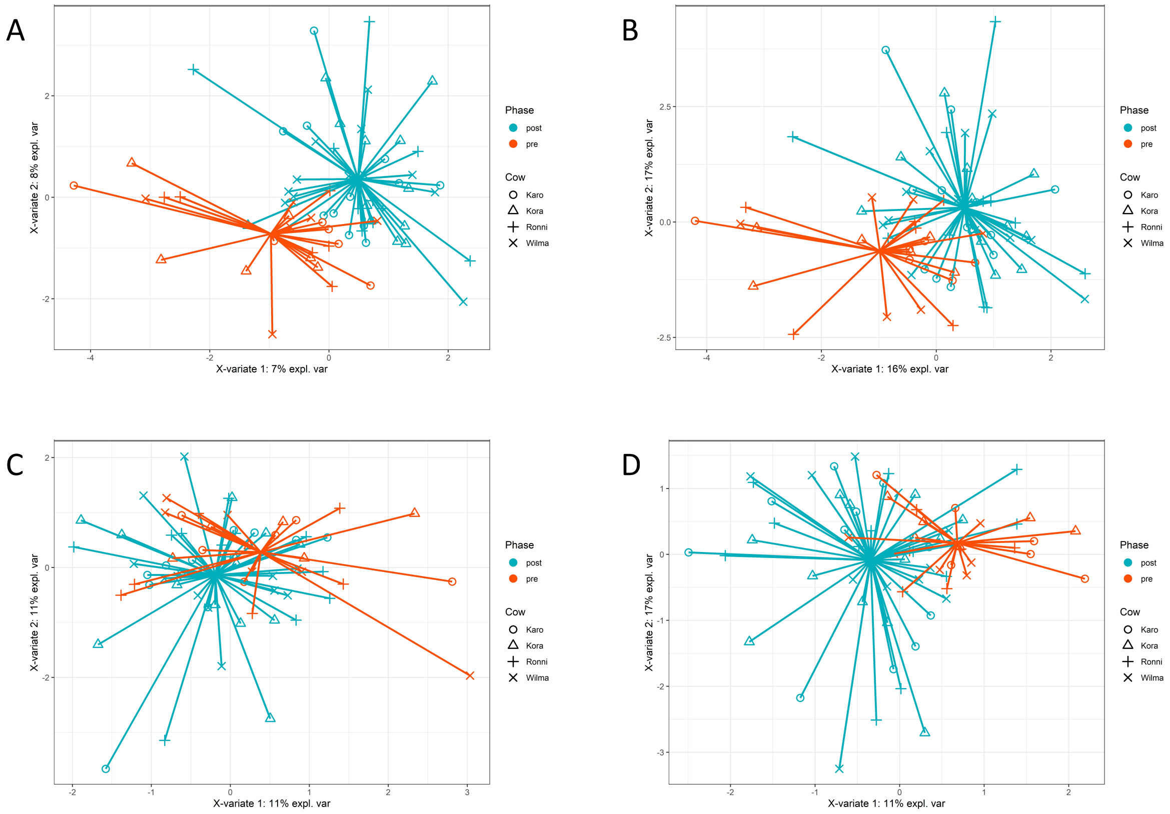

Supplementary Figure S17. Supervised Clustering of the results of Bovalto Respi 3 treatment (group A), three components with a maximum of five features each. A: sPLS-DA using all parameters (AUROC: “pre” vs “post”: 0.959); B: sPLS-DA using blood parameters (AUROC: “pre” vs “post”: 0.929); C: sPLS-DA using milk parameters (AUROC: “pre” vs “post”: 0773); D: sPLS-DA using the parameters analyzed by external laboratory (AUROC: “pre” vs “post” 0.843).

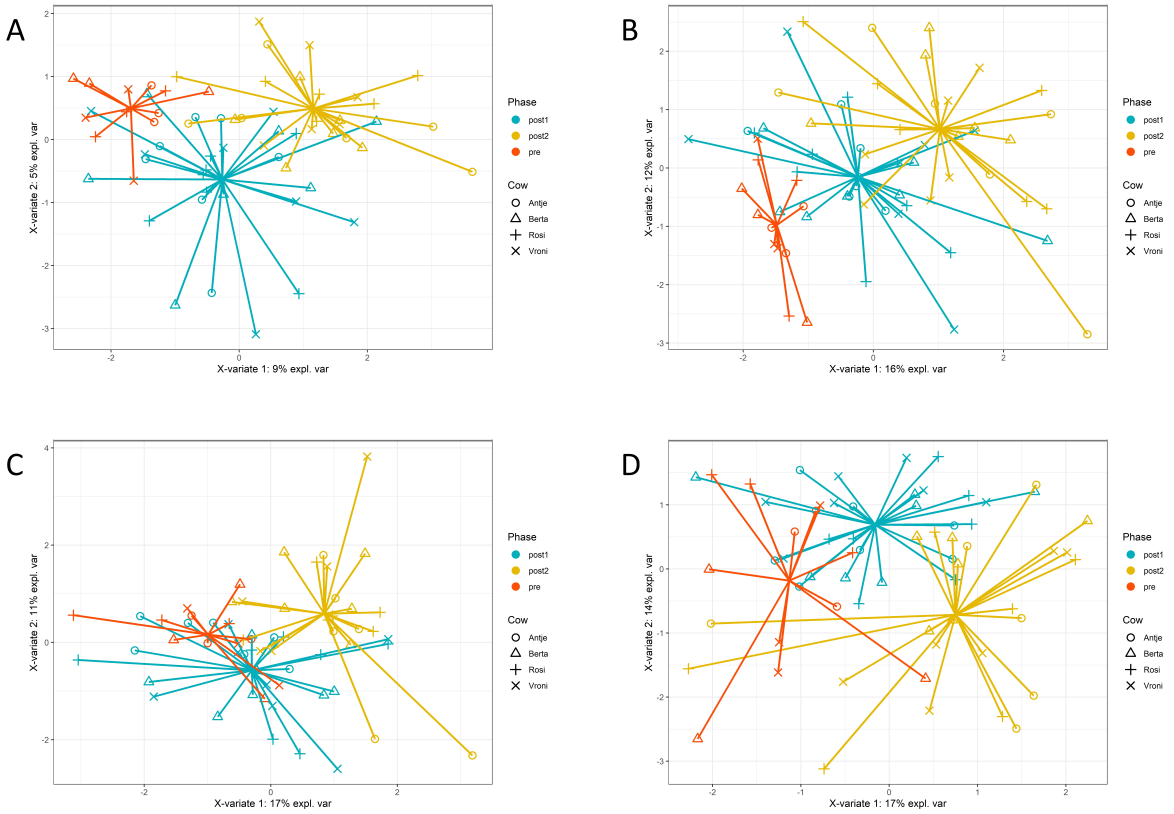

Supplementary Figure S18. Supervised Clustering of the results of Insol Trichophyton treatment (group B), three components with a maximum of five features each. A: sPLS-DA using all parameters (AUROC: “pre” vs others: 0.989, “post1” vs others: 0.876, “post2” vs others: 0.999); B: sPLS-DA using blood parameters (AUROC: “pre” vs others: 0.976, “post1” vs others: 0.737, “post2” vs others: 0.938); C: sPLS-DA using milk parameters (AUROC: “pre” vs others: 0.880, “post1” vs others: 0.837, “post2” vs others: 0.921); D: sPLS-DA using the parameters analyzed by external laboratory (AUROC: “pre” vs others 0.886, “post1” vs others: 0.807, “post2” vs others: 0.951).
